# Multi‐ancestry meta‐analysis identifies genetic modifiers of age‐at‐onset of Alzheimer's disease at known and novel loci

**DOI:** 10.1002/alz.70489

**Published:** 2025-08-29

**Authors:** Elizabeth E. Blue, Jai Broome, Diane Xue, Hanley Kingston, Nicola H. Chapman, Stephanie Gogarten, Alzheimer's Disease Genetics Consortium (ADGC), Adam C. Naj, Ellen M. Wijsman

**Affiliations:** ^1^ Department of Medicine, Division of Medical Genetics University of Washington Seattle Washington USA; ^2^ Institute for Public Health Genetics University of Washington Seattle Washington USA; ^3^ Brotman Baty Institute University of Washington Seattle Washington USA; ^4^ Department of Medicine Division of General Internal Medicine University of Washington Seattle Washington USA; ^5^ Department of Genetics, Perelman School of Medicine University of Pennsylvania Philadelphia Pennsylvania USA; ^6^ San Mateo Public Health Laboratory San Mateo California USA; ^7^ Department of Biostatistics University of Washington Seattle Washington USA; ^8^ Department of Biostatistics, Epidemiology, and Informatics, Perelman School of Medicine University of Pennsylvania Philadelphia Pennsylvania USA; ^9^ Penn Neurodegeneration Genomics Center, Department of Pathology and Laboratory Medicine Perelman School of Medicine University of Pennsylvania Philadelphia Pennsylvania USA; ^10^ Department of Genome Sciences University of Washington Seattle Washington USA

**Keywords:** age at onset, apolipoprotein E, diversity, multi‐ancestry, sex differences

## Abstract

**INTRODUCTION:**

Much of Alzheimer's disease (AD) risk is explained by age, apolipoprotein E (*APOE*) genotype, and sex. We sought to identify genetic modifiers of age at onset (AAO) of AD while probing the influence of sex and *APOE* among those with diverse ancestry.

**METHODS:**

We performed genome‐wide association studies (GWASs) of AAO in two diverse samples followed by meta‐analysis, contrasting results with and without adjustment for sex and *APOE*. Genome‐wide significance was set to *p* < 5×10^−8^.

**RESULTS:**

GWASs adjusting for sex, *APOE*, population structure, and relatedness revealed 17 significant loci including independent associations at AD risk loci and four novel signals. *APOE* adjustment influenced GWAS effect sizes across the genome while sex adjustment had minimal effect.

**DISCUSSION:**

We identified association signals within a diverse but relatively small sample, replicating loci recently discovered in large European ancestry‐only GWASs, and illustrated the power of using a quantitative trait like AAO over a binary diagnosis trait.

**Highlights:**

- Survival analysis approach identified known and novel genetic modifiers of Alzheimer's disease (AD).
- Multi‐ancestry analyses revealed independent signals at known AD loci.
- Apolipoprotein E adjustment influenced variant effects across the genome.

## 1 BACKGROUND

Age, sex, and apolipoprotein E (*APOE*) genotype are major risk factors for Alzheimer's disease (AD). The annual incidence of AD increases 19‐fold between the ages of 65 to 70 and 85+ years; the prevalence of AD is one third higher in women than men;^1^ and *APOE* genotype, specifically elevated AD risk with ε4 and the protective effect of ε2, can explain nearly a quarter of AD heritability.^2^ Despite this, genome‐wide association studies (GWASs) for AD do not consistently address the effects of age, sex, and *APOE* genotype in their modeling approaches. This inconsistency complicates the interpretation of meta‐analyses combining results across outcomes and models^3^, ^4^ and reduces statistical power to detect novel AD susceptibility loci.

While age at onset (AAO) of AD is highly heritable (57%–78%),^5^, ^6^ relatively few GWASs have considered it as an outcome.^2^, ^7^, ^8^, ^9^, ^10^, ^11^, ^12^, ^13^, ^14^ Analyzing AAO as a censored trait offers improved statistical power relative to a typical case/control design by leveraging age information not only from those affected by the disease but also those who appear protected from AD at older ages.^15^, ^16^, ^17^ Despite their smaller sample sizes, AAO GWASs have replicated 20 AD risk loci and nominated three novel signals near genes associated with molecular or neuropathological changes in AD.^2^, ^7^, ^8^, ^9^, ^10^, ^11^, ^12^, ^13^, ^14^ This suggests that AAO of AD and AD risk share an overlapping, but not necessarily identical, genetic architecture.

Most of the > 150 GWASs of AD risk or AAO represent non‐Hispanic White participants^18^, ^19^ while other populations remain underrepresented, with underpowered sample sizes unable to detect association signals with comparable effect sizes.^20^ Many AD risk loci identified in European ancestry–‐focused GWASs are nominally associated with AD in Black Americans (*APOE*, *ABCA7*, *BIN1*, *CASS4*, *CD33*, *CELF*, *CR1*, *BIN1*, *EPHA1*, *NME8*),^21^, ^22^ Caribbean Hispanics (*BIN1*, *CLU*, *Picalm*),^23^ and East Asians (*ABCA7*, *BIN1*, *CD33*, *CNTNAP2*, *PICALM*, *SORL1*).^22^, ^24^, ^25^ Many variants associated with AD vary substantially in frequency across continental populations^26^ due to the history of human migration (e.g., *APOE* ^27^). Haplotypic variation at those loci influences effect size estimates^28^ and hampers our ability to generalize GWAS results and estimate polygenic risk of disease.^29^, ^30^, ^31^ Local ancestry differences capturing this haplotypic variation have been significantly associated with AD risk near *ABCA7*, *CD33*, and *GRINB3* in Blacks^32^ and at *APOE* in Hispanics.^33^, ^34^ Multi‐ancestry analyses offer an alternative strategy to identify genetic variation associated with AD phenotypes while better capturing human diversity^22^, ^35^, ^36^ and offer results that improve the generalizability of polygenic risk scores for AD.^37^

Here, we perform a GWAS for AAO of AD in a diverse set of AD cases and controls in both a discovery and replication data set, then meta‐analyze results to detect shared signals. Given the relationships among *APOE*, ancestry, and AD risk,^19^, ^33^, ^34^, ^38^ we explore the effects of *APOE* adjustment on association signals. We find shared genetic architecture between AD risk and AAO loci, novel loci associated with AAO of AD, and evidence that *APOE* adjustment alters the association between AAO of AD and variants across the genome.

## 2 METHODS

### 2.1 The discovery data set

We gathered controlled‐access genotype array and phenotype data through approved applications to the database of Genotypes and Phenotypes (dbGaP; study accession: phs00496) and the National Institute on Aging Genetics of Alzheimer's Disease Data Storage Site (NIAGADS; study accessions NG00020, NG00022‐NG00024, NG00026, NG00028‐NG00031, NG00034, NG00047, NG00068‐NG00071). Study‐specific sample quality control (QC) required missing < 5% of genotypes as well as pedigree^39^ and sex checks^40^ to exclude sample swaps. We restricted analysis to those with AD case or control status (excluding mild cognitive impairment), AAO for cases and age ‐at ‐last ‐evaluation (AAE) data for controls, and both sex and *APOE* genotype data. Where data were available, outliers were excluded where age at death was > 10 years greater than AAO. *APOE* covariate adjustment used imputed ε2 and ε4 allele dosages to account for uncertainty in genotyping. Samples with discordant observed versus imputed sex or *APOE* genotype were excluded. When selecting which duplicate sample to exclude, we prioritized those missing phenotype data, then by genotype missingness rate, and finally selected one at random if they otherwise did not differ. Study‐specific autosomal variant QC included genotype completeness ≥ 95%, minor allele frequency (MAF) > 5%, Hardy–Weinberg equilibrium^41^ *p* < 0.01 using the Robust Unified Hardy–Weinberg Equilibrium Test to accommodate population structure within a study, and unambiguous genotypes. Genotypes were aligned to hg19, phased by Eagle v2.4,^42^ then imputed^43^ to the Trans‐Omics for Precision Medicine (TOPMed)^44^ reference panel aligned to hg38 using minimac4^45^ as implemented in the TOPMed Imputation Server.^43^ Study‐specific imputation QC included average call rate ≥ 0.95 and *r* ^2^ ≥ 0.3 for common variants with MAF ≥ 0.05 or *r* ^2^ ≥ 0.5 for uncommon variants with MAF < 0.05. Study‐specific imputed genotypes and phenotype data were combined into a single data set. Imputed variants with MAF < 1% or missing ≥ 5% of genotypes in the combined data set were excluded. Samples with missing or discordant observed and imputed ε2/ε3/ε4 genotypes were excluded from *APOE*‐adjusted GWAS.

**RESEARCH IN CONTEXT**

1. **Systematic review**: The authors performed a literature review of published articles describing genome‐wide association studies of Alzheimer's disease (AD) and age at onset (AAO). Previous studies offer evidence that the association between genotype and AD can vary dramatically in strength, direction of effect, or both when studies vary by major risk factors including age, sex, and ancestry.
2. **Interpretation**: Our multi‐ancestry genome‐wide association study of AAO of AD identified shared genetic architecture with AD risk, including novel association signals and independent signals at established AD risk loci. Apolipoprotein E adjustment influenced variant effects genome wide, consistent with a strong relationship with both AD risk and human population structure.
3. **Future directions**: Future studies of genome sequence data in independent data sets representing similar levels of population diversity are needed to validate and fine‐map these associations. Functional studies in appropriate cell types are needed to test causal relationships among prioritized variants, genes, and AD.

### 2.2 The replication data set

The Alzheimer's Disease Genetics Consortium (ADGC) data were imputed to the TOPMed reference as previously described.^35^ Data were shared as study‐specific cohorts defined by reported race and ethnicity. We selected a subset of this ADGC data to minimize overlap with the discovery data (ACT‐AA, ACT2, ACT3, ACT3‐AA, ACT3‐Asian, ACT3‐Hispanic, ADC1‐2‐AA, ADC10, ADC10‐AA, ADC10‐Asian, ADC10‐Hispanic, ADC11, ADC11‐AA, ADC11‐Asian, ADC11‐Hispanic, ADC12, ADC12‐AA, ADC12‐Asian, ADC12‐Hispanic, ADC3‐AA, ADC8, ADC8‐AA, ADC8‐Hispanic, ADC9, ADC9‐AA, ADC9‐Hispanic, ADNI, BIOCARD, CHAP‐AA, CHAP2, CHOP‐AA, EAS, GSK, MAYO, MIRAGE300‐AA, MIRAGE600‐AA, NBB, NIALOAD‐NCRAD‐AA, OHSU, PRADI‐Hispanic, REAAADI‐AA, RMayo, TARCC1, TARCC3, TARCC3‐AA, TARCC3‐Hispanic, TARCC4‐Hispanic, UKS, UMVUTARC2, WASHU2, WHICAP). Study‐specific imputation QC included average call rate ≥ 0.95 and *r* ^2^ ≥ 0.3 for common variants with MAF ≥ 0.05 or *r* ^2^ ≥ 0.5 for uncommon variants with MAF < 0.05. Study‐specific imputed genotypes and phenotype data were combined into a single data set. Sample QC included missing < 5% of genotypes and restricted analysis to those with AD case or control status (excluding mild cognitive impairment), AAO for cases or AAE for controls, and both sex and *APOE* genotype data. *APOE* covariate adjustment used imputed allele dosages to account for uncertainty in genotyping. Samples with discordant observed versus imputed sex or *APOE* genotype were excluded. Imputed variants with MAF < 0.5% or missing ≥ 5% genotypes in the combined data set were excluded.

### 2.3 Human subjects

This study was approved by the University of Washington Human Subjects Division (STUDY00000240) and was performed in accordance with ethical standards consistent with the 1964 Declaration of Helsinki. This study included data from participants across demographic boundaries including reported sex, race, and ethnicity. As described below, analyses adjusted for relatedness and population structure within the data, improving the generalizability of our results.

### 2.4 Relatedness and population structure

Relatedness and principal components (PC) analysis was performed on imputed genotypes after QC, filtering variants for MAF > 5%. For each data set, relatedness was initially estimated by KING‐robust^39^ after pruning variants for linkage disequilibrium (*r* ^2^ < √0.01). Duplicate samples were excluded, prioritizing first those missing phenotype data, then higher genotyping rate, and finally selecting one at random if they otherwise did not differ. PCs accounting for relatedness were first estimated using PC‐AiR^46^ then relatedness estimates were recalibrated by PC‐Relate^47^ and then these estimates were used for another round of PC‐AiR/PC‐Relate to orthogonally partition PCs and the genetic relatedness matrix (GRM). The most informative PCs were selected using the inflection point of scree plots as well as clustering within pairwise PC plots. To speed GWAS, we used a sparse GRM, setting < fourth‐degree relatedness to zero.

### 2.5 Association testing

GWAS analyses were restricted to imputed genotypes with MAF ≥ 1% and AAO or AAE. The phenotype was defined as the Martingale residuals from a Cox proportional hazards analysis performed using the survival package in R (v3.5), using AAO of AD for cases and AAE for controls, adjusted for sex, imputed ε2 dosage, imputed ε4 dosage, and the most informative PCs. Using the LMM‐OPS framework,^48^ we tested the association between imputed genotypes and this phenotype, adjusting for the most informative PCs with fixed effects and the GRM with random effects.

We performed two secondary GWASs to evaluate the sensitivity to covariate adjustment. The first alternative GWAS mimicked the original GWAS but removed *APOE* covariates, while the second removed both *APOE* and sex covariates.

Genomic inflation^49^ in the discovery and replication GWAS was measured using λ and linkage disequilibrium (LD) score regression intercepts.^50^ Results from the discovery and replication GWAS were meta‐analyzed using Han and Eskin's Random Effects model (RE2) implemented in Metasoft^51^ and correcting for genomic inflation of mean effects. Genome‐wide significance was defined as *p* < 5×10^−8^, and Manhattan plots were drawn in R.^52^

### 2.6 Interpreting association signals

Hazard ratios for individual markers were estimated by including the marker as a covariate in the same Cox proportional hazards test used to define the associated outcome. The genomic context of GWAS signals was plotted using the locuszoomr (v0.3.5) R package, annotated with LD information from 1000 Genomes Europeans^53^, ^54^ provided by the LDlinkR R package (v1.4.0)^55^ and gene annotations from Ensembl v113^56^ provided by the AnnotationHub R package (v3.14.0). Correlation and differences in effect sizes were calculated and illustrated in R.

We sought evidence of replication for loci previously associated with AD risk or AAO of AD. We extracted the lead marker from genome‐wide significant associations with AD risk across 13 major GWASs for AD or proxy AD phenotypes^57^ and study‐wide significant associations with AAO^2^, ^7^, ^8^, ^9^, ^10^, ^11^, ^12^, ^13^, ^14^ outside the *APOE* region (chr19:43,905,796‐45,909,393; hg38). We selected one marker per locus, prioritizing first by strength of evidence of replication, then by effect size, and finally by *p* value. Because *APOC1* is near *APOE*, we performed a separate search of the GWAS catalog^18^ for “*APOC1*” and reviewed all listings with the reported trait “Alzheimer's disease.”

Variants were connected to target genes using Open Targets Genetics,^58^, ^59^ https://genetics.opentargets.org/, last updated October 2022. If a variant fell at a known AD GWAS locus, annotation was restricted to the nominated gene for that locus; otherwise, the top candidate was selected based on variant to function (V2F) support. Phenome‐wide association study (PheWAS) data were extracted from the NHGRI/EMBL‐EBI GWAS Catalog,^18^ restricted to those with study‐specific *p* < 5×10^−8^. Genes were connected to AD biological domains^60^ and therapeutic targets using Agora, https://agora.adknowledgeportal.org/, site v3.4.0 and data version syn13363290‐v68. A subset of variants was annotated with allele frequencies in 1000 Genomes/Human Genome Diversity Project (HGDP) reference populations.^53^

## 3 RESULTS

### 3.1 Diversity within discovery and replication data sets

**FIGURE 1:**
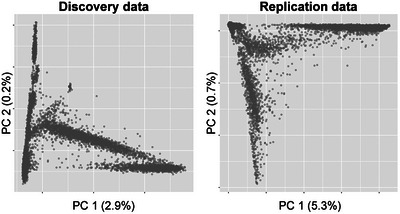
Genetic diversity within the discovery and replication data sets. X axis: the first principal component (PC). Y axis: the second PC. Each PC is labeled by the amount of genetic variance explained.

After QC, the discovery and replication data sets were similarly balanced between cases and controls by sample sizes, age distributions, sex, and *APOE* genotype frequencies (Table 1). *APOE* genotype mismatch rates were similar in the discovery and replication sets for ε2 (1.13% vs. 1.87%) while the ε4 mismatch rate was greater in the replication data set (2.29% vs. 5.23%). Sample‐level QC for missing data in the replication data demonstrated a disproportionate effect on cohorts reporting Asian or Black race. Both the discovery and replication data sets are enriched for non‐Hispanic White participants (72% and 63%, respectively), with higher proportions of Hispanic ethnicity and lower proportions of Black race in the discovery data. The discovery data showed relative enrichment of non‐Hispanic White cases and Black controls, but this pattern was less evident in the replication data. PC analysis (PCA) within each data set reveals complex patterns of genetic variation (Figure 1), corresponding to human population structure rather than technical artifacts (Figures S1 and S2 in supporting information).

**TABLE 1:** Sample description.

	Discovery (*n* = 22,044)		Replication (*n* = 19,483)	
*n*	Controls	Cases	Controls	Cases
Males	4197	4484	4417	2930
Females	6963	6400	7938	4198
**Age (years)**				
Mean	76.3	73.3	74.5	72.5
Min	32	30	43	33
Max	103	102	108	107
Median	77	74	74	73
***APOE* frequencies**				
ε2	7.2%	3.5%	7.5%	3.9%
ε4	15.2%	36.4%	15.6%	34.8%
**Reported race/ethnicity**				
Non‐Hispanic White	63.5%	78.7%	61.4%	66.7%
Black	13.5%	3.0%	28.2%	22.9%
Hispanic	22.9%	17.9%	10.4%	10.5%
Other values	0.1%	0.4%	0.0%	0.0%

*Notes*: Age: age ‐at onset of Alzheimer's disease for cases, age at last evaluation or visit for controls. *APOE* frequencies and reported race/ethnicity proportions represent only non‐missing values. “Other” values include American Indian/Alaskan Native, Asian/Pacific Islander, and Other demographic categories.

Abbreviation: *APOE*, apolipoprotein E.

### 3.2 GWAS for AAO of AD with both sex and *APOE* adjustment

**FIGURE 2:**
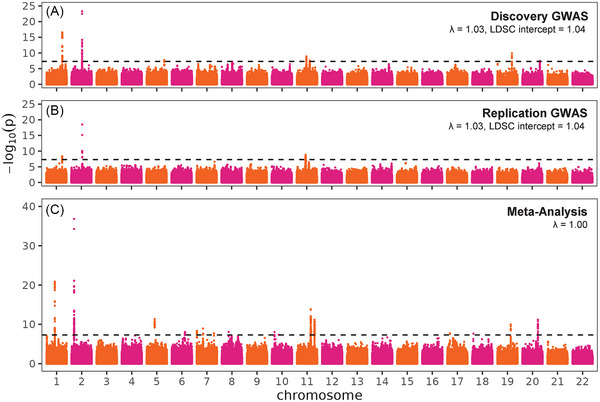
GWAS for AAO of AD, adjusting for sex, ε2, ε4, population structure, and relatedness. A, GWAS in the discovery data. B, GWAS in the replication data. C, Meta‐analysis of discovery and replication GWASs, adjusted for genomic inflation. AAO, age at onset; AD, Alzheimer's disease; GWAS, genome‐wide association study; LDSC, linkage disequilibrium score regression.

The discovery GWAS of 9,111,557 variants and 21,736 participants adjusted for sex and *APOE* ε2 and ε4 dosages identified significant evidence of association between AAO of AD and seven known AD risk loci near *APOE, BIN1, CASS4, CR1, HAVCR2, MS4A4A*, and *PICALM* (λ = 1.03, LD score intercept = 1.04; Figure 2A, Table S1 in supporting information). The replication analysis of 8,634,132 variants and 19,483 participants supported each of these signals while no other loci reached genome‐wide significance (λ = 1.03, LD score intercept = 1.04; Figure 2B, Table S1). Meta‐analysis of the discovery and replication GWAS identified 16 genome‐wide significant associations (λ = 1), including 14 known AD risk loci near *APOE* (*APOC1*), *BIN1*, *CASS4*, *CD2AP*, *CR1*, *ECHDC3* (*LOC105376412*), *EPHA1, HAVCR2* (*ADRA1B*)*, MS4A4A*, *MYO15A* (*FBXW10*)*, PICALM*, *pILRA* (*NYAP1*), *SHARPIN*, and *UMAD1* while adding two new signals: chromosomes 11q13.1 near *CATSPERZ* and 18p11.21 near *LDLRAD4* (Figure 2C, Table 2). Most of these signals had robust support from both the discovery and the replication GWASs (2q14.3 *BIN1*, 6p12.3 *CD2AP*, 7p21.3 *UMAD1*, 7q22.1 *NYAP1*, 7q34‐q35 *EPHA1*, 8q24.3 *SHARPIN*, 10p14 *LOC105376412*, 11q12.2 *MS4A4A*, 11q14.2 *PICALM*, 20q13.31 *CASS4*) though two showed some attenuation of effect in the replication study (1q32.2 *CR1*, 19q13.32 *APOC1*; Table S2 in supporting information). Four signals had both nominally significant evidence of heterogeneity and opposing directions of effect (5q33.3 *ADRA1B*, 11q13.1 *CATSPERZ*, 17p11.2 *FBXW10*, 18p11.21 *LDLRAD4*).

**TABLE 2:** Genome‐wide significant loci in meta‐analysis of GWAS for AAO of AD, adjusting for sex, *APOE* genotypes, population structure, and relatedness.

			Discovery				Replication				Meta‐analysis				
Locus	Gene	Variant	AAF	β	SE	*p*	AAF	β	SE	*p*	β	SE	*p*	*I* ^2^ *(%)*	Q *p*
1q32.2	*CR1*	1:207623552:A:T	0.817	−0.078	0.009	6.68E‐17	0.846	−0.051	0.009	1.69E‐08	−0.064	0.014	1.35E‐21	77.30	3.58E‐02
2q14.3	*BIN1*	2:127135234:C:T	0.404	0.073	0.007	3.16E‐23	0.405	0.058	0.007	3.04E‐19	0.065	0.007	1.52E‐37	52.95	1.45E‐01
5q33.3	*HAVCR2*	5:159951499:G:A	0.024	−0.130	0.023	1.85E‐08	0.031	0.044	0.018	1.53E‐02	−0.042	0.087	4.43E‐12	97.15	3.21E‐09
6p12.3	*CD2AP*	6:47586441:G:A	0.255	0.035	0.008	1.67E‐05	0.242	0.031	0.007	2.77E‐05	0.033	0.005	8.70E‐09	0.00	7.32E‐01
7p21.3	*UMAD1*	7:7816063:G:A	0.555	−0.035	0.007	9.92E‐07	0.538	−0.022	0.006	4.74E‐04	−0.028	0.006	1.87E‐08	41.97	1.89E‐01
7q22.1	*pILRA*	7:100494172:T:C	0.729	0.039	0.008	1.34E‐06	0.748	0.029	0.007	1.25E‐04	0.033	0.005	4.81E‐09	0.00	3.59E‐01
7q34‐q35	*EPHA1*	7:143435090:G:A	0.567	0.027	0.007	1.66E‐04	0.524	0.034	0.007	2.49E‐07	0.031	0.005	1.11E‐09	0.00	5.13E‐01
8q24.3	*SHARPIN*	8:144103704:G:A	0.080	0.067	0.014	2.49E‐06	0.065	0.054	0.014	1.51E‐04	0.061	0.010	8.24E‐09	0.00	5.13E‐01
10p14	*ECHDC3*	10:11678309:A:G	0.358	0.032	0.008	2.19E‐05	0.331	0.030	0.007	2.21E‐05	0.031	0.005	8.78E‐09	0.00	8.26E‐01
11q12.2	*MS4A6A*	11:60251788:G:GTA	0.334	−0.042	0.008	5.39E‐08	0.293	−0.043	0.007	3.59E‐09	−0.042	0.005	1.35E‐14	0.00	9.43E‐01
11q13.1	*CATSPERZ*	11:64301284:T:C	0.933	0.034	0.017	4.75E‐02	0.856	−0.060	0.011	1.20E‐07	−0.014	0.047	7.18E‐09	95.15	5.57E‐06
11q14.2	*PICALM*	11:86120648:A:G	0.679	0.039	0.008	3.79E‐07	0.704	0.035	0.007	4.23E‐07	0.037	0.005	6.44E‐12	0.00	7.67E‐01
17p11.2	*MYO15A*	17:18737894:G:A	0.029	−0.065	0.024	5.94E‐03	0.024	0.104	0.024	1.30E‐05	0.019	0.085	1.88E‐08	96.05	4.93E‐07
18p11.21	*LDLRAD4*	18:13585011:C:T	0.911	0.043	0.012	4.47E‐04	0.916	−0.043	0.012	2.46E‐04	0.000	0.043	2.34E‐08	96.11	3.94E‐07
19q13.32	*APOC1*	19:44916825:A:C	0.184	0.095	0.015	1.31E‐10	0.137	0.030	0.013	2.43E‐02	0.062	0.033	1.02E‐10	90.76	1.01E‐03
20q13.31	*CASS4*	20:56414777:T:A	0.095	−0.064	0.012	1.35E‐07	0.112	−0.050	0.010	8.88E‐07	−0.056	0.008	6.77E‐12	0.00	3.82E‐01

*Notes*: Variant: Chromosome: Position on hg38: Reference allele: Alternate allele; β: Effect size for the alternate allele; Q statistic *p* value: Evidence for heterogeneity between our discovery and replication studies.

Abbreviations: AAF, alternative allele frequency; AAO, age at onset; AD, Alzheimer's disease; *APOE*, apolipoprotein E; GWAS, genome‐wide association study; SE, standard error of effect size.

#### 3.2.1 Evidence supporting loci with significant heterogeneity

**FIGURE 3:**
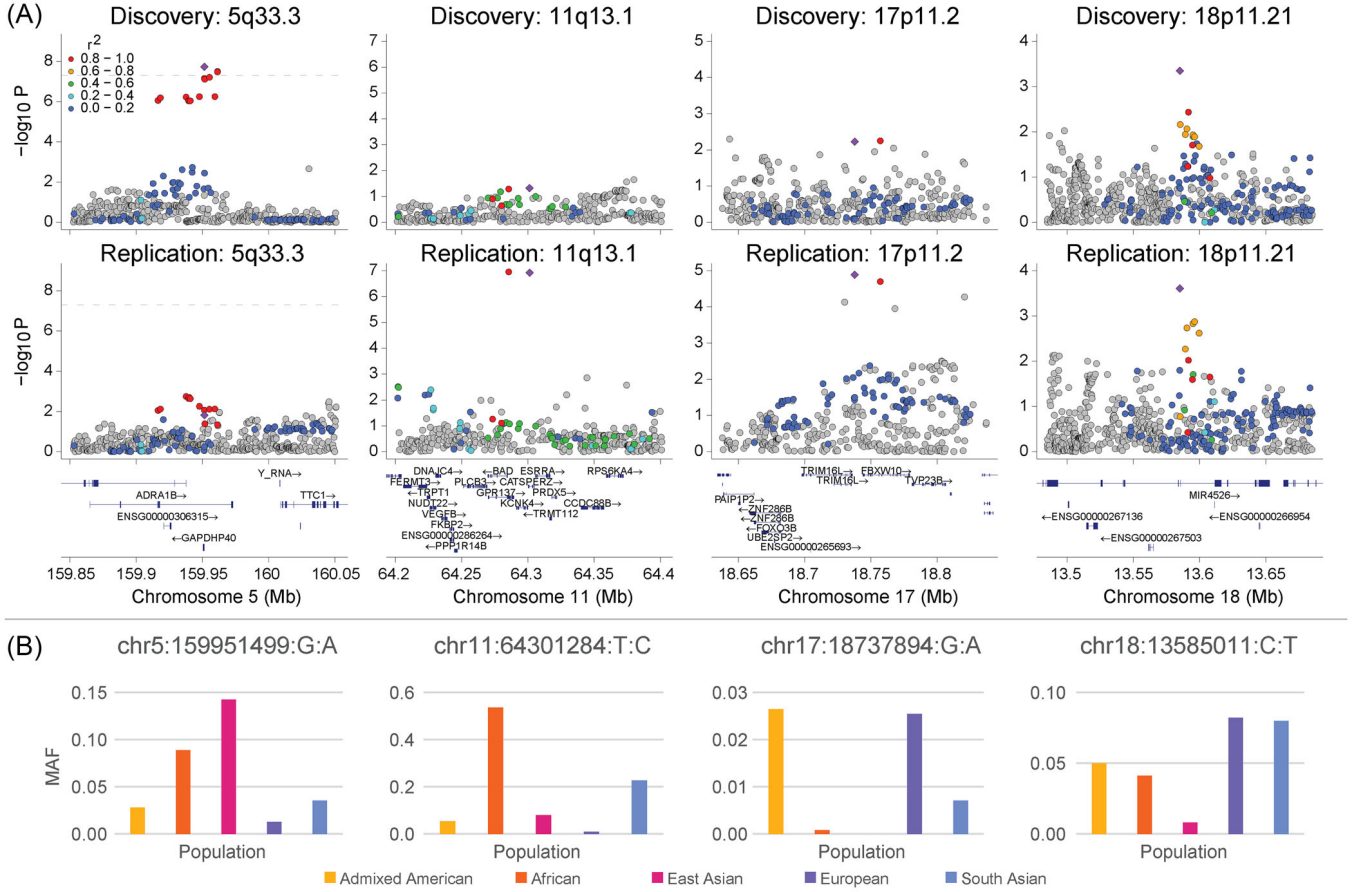
Support for heterogeneous meta‐analysis signals. A, Lead variant from meta‐analysis is shown as a purple diamond. Linkage disequilibrium information is specific to 1000 Genomes European samples. Gene labels were restricted to protein coding genes for 11q13.1. B, Minor allele frequency (MAF) variation across 1000 Genomes reference populations. Variants are presented as chromosome:position:reference allele:alternate allele. Reference populations are color coded as gold: admixed Americans; orange: African; magenta: East Asian; purple: European; blue: South Asian.

We investigated the support for each genome‐wide significant locus in the meta‐analysis with strong evidence for heterogeneity and effect size estimates in opposite directions between the discovery and replication data sets. These variants were typically uncommon in both data sets (MAF < 10%), though the lead single nucleotide polymorphism (SNP) at 11q13.1 reached nearly 15% in the replication data. Two of these signals are driven by a strong signal in a single data set (5q33.3 and 11q13.1) with only nominally significant support for an opposing direction of effect in the other data set (*p* < 0.05). The evidence supporting the other two heterogeneous signals (17p11.2 and 18p11.21) was more modest and more evenly distributed between data sets. LocusZoom plots reveal that each signal, when evident, is supported by multiple variants in a haplotype (Figure 3A). Variation in sample demographics and variation in MAFs across reference populations suggest that the underlying haplotypic variation may vary between our discovery and replication data sets (Figure 3B).

#### 3.2.2 Relationship with AD risk alleles

To better understand the relationship between our association signals and prior AD GWAS, we compared our evidence of association between AAO of AD and the lead SNP for an AD GWAS signal from the literature^57^ (Table 3). Three of our lead SNPs are the most frequently reported lead SNP at the locus across recent AD GWASs: *BIN1*, *ECHDC3*, and *SHARPIN1* (Figures S3–S5 in supporting information). While our lead SNPs at 1q32.2 (*CR1*), 7p21.3 (*UMAD1*), and 11q12.2 (*MS4A6A*) differed, they were in high LD with the reported SNPs at those loci (*r* ^2^ > 0.8) which reached genome‐wide significance in our study (*p* < 5 × 10^−8^, Figures S6–S8 in supporting information). Our GWAS refines the known haplotypes associated with AD on 6p12.3 (*CD2AP*), 7q22.1 (*pILRA*), 11q14.2 (*PICALM*), and 20q13.31 (*CASS4*), with lead SNPs in moderate LD with the reported lead SNPs with attenuated *p* values in our study (Figures S9–S12 in supporting information). However, our analysis does not support the association between the reported SNPs near *EPHA1*, *HAVCR2*, and *MYO15A* (*p* > 0.05, Figures 3 and S13 in supporting information), suggesting that our signals at 5q33.3, 7q34‐q35, and 17p11.2 are independent. As ε4 dosage is included in the GWAS model it is not significantly associated with AAO of AD in this study, though the *APOC1* variant on 19q13.32 is on the same haplotype in both the discovery and replication data sets (Figure S14 in supporting information). While *APOC1* SNPs have been associated with AD, our literature review found no prior genome‐wide significant signal at *APOC1* that was independent of the *APOE* ε2 and ε4 alleles.

**TABLE 3:** Comparison of lead variants at known AD GWAS loci.

										Discovery		Replication	
Locus	Gene	Lead variant	HR	95% CI	*p*	Known SNP	HR	95% CI	*p*	*r* ^2^	D'	*r* ^2^	D'
1q32.2	*CR1*	rs10863417	0.94	0.91‐0.96	1.35E‐21	rs679515	0.94	0.91‐0.97	2.57E‐21	0.96	0.99	0.65	0.99
2q14.3	*BIN1*	rs6733839	1.07	1.05‐1.08	1.52E‐37	rs6733839	–	–	–	–	–	–	–
5q33.3	*HAVCR2*	rs62377696	0.96	0.81‐1.14	4.43E‐12	rs6891966	1.01	0.99‐1.02	4.02E‐01	0	0.03	0	0.15
6p12.3	*CD2AP*	rs9395285	1.03	1.02‐1.04	8.70E‐09	rs9381563	0.98	0.97‐0.99	2.49E‐04	0.28	0.99	0.13	0.99
7p21.3	*UMAD1*	rs10276423	0.97	0.96‐0.98	1.87E‐08	rs6943429	0.97	0.96‐0.99	1.87E‐08	0.89	0.96	0.87	0.95
7q22.1	*PILRA*	rs12539172	1.03	1.02‐1.05	4.81E‐09	rs1859788	1.03	1.02‐1.04	1.18E‐08	0.32	0.92	0.34	0.88
7q34‐q35	*EPHA1*	rs9640386	1.03	1.02‐1.04	1.11E‐09	rs10808026	0.99	0.97‐1.01	1.17E‐01	0	0.02	0	0.09
8q24.3	*SHARPIN*	rs34173062	1.06	1.04‐1.08	8.24E‐09	rs34173062	–	–	–	–	–	–	–
10p14	*ECHDC3*	rs7920721	1.03	1.02‐1.04	8.78E‐09	rs7920721	–	–	–	–	–	–	–
11q12.2	*MS4A6A*	rs3041800	0.96	0.95‐0.97	1.35E‐14	rs1582763	0.96	0.95‐0.97	1.59E‐14	0.66	1	0.69	1
11q14.2	*PICALM*	rs472486	1.04	1.03‐1.05	6.44E‐12	rs3851179	1.03	1.02‐1.05	1.78E‐10	0.42	0.85	0.42	0.82
17p11.2	*MYO15A*	rs149428166	1.02	0.86‐1.20	1.88E‐08	rs2242595	0.99	0.97‐1.01	6.38E‐02	0	0.37	0	0.89
19q13.32	*APOC1*	rs73052335	1.06	1.00‐1.13	1.02E‐10	rs429358	1.06	0.68‐2.56	4.51E‐01	0.27	0.93	0.36	0.91
20q13.31	*CASS4*	rs6014722	0.95	0.93‐0.96	6.77E‐12	rs6014724	0.96	0.93‐0.98	1.25E‐07	0.54	0.87	0.53	0.75

*Notes*: Our meta‐analysis results for both the lead variant in this study and the lead SNP from prior AD GWASs are compared, along with their pairwise estimates of linkage disequilibrium within our discovery and replication data sets. Known SNP, Lead variant in prior AD GWAS; *r* ^2^, D': Measures of linkage disequilibrium between the lead variants in this study and prior AD GWAS.

Abbreviations: AD, Alzheimer's disease; CI, confidence interval; GWAS, genome‐wide association study; gene: Nominated gene at AD GWAS locus; HR, hazard ratio; SNP, single nucleotide polymorphism.

### 3.3 Covariate effects

We explored whether differences in model specification might explain the differences between this study and the recent AD GWAS literature focused on case–control status and non‐Hispanic White samples.^3^, ^4^, ^61^, ^62^, ^63^, ^64^, ^65^, ^66^, ^67^, ^68^ Prior GWASs of AD cohorts with clinical/pathological diagnoses typically included age and sex as covariates,^61^, ^62^ whereas GWASs including proxy AD phenotypes (e.g., parental history of dementia) in biobank datasets typically did not adjust for age^3^, ^64^, ^67^ or sex^3^, ^64^, ^67^ or did so inconsistently.^4^, ^65^, ^68^ These proxy‐GWAS also typically meta‐analyzed their results with GWAS summary statistics from studies that included age and/or sex as covariates. None included *APOE* genotype as a covariate.

#### 3.3.1 Main covariate effects

Each covariate was strongly associated with AAO of AD after adjustment for the first nine PCs, with both data sets offering similar effect size estimates. Female sex was associated with reduced hazard of AD (discovery hazard ratio [HR]: 0.88 [95% confidence interval (CI): 0.84–0.91], *p* = 2.75 × 10^−11^; replication HR: 0.87 [95% CI: 0.83–0.91], *p* = 1.97 × 10^−08^). Dose of the *APOE* ε2 allele was associated with a strong protective effect (discovery HR: 0.66 [95% CI: 0.61–0.71], *p* < 2 × 10^−16^; replication HR: 0.68 [95% CI: 0.63–0.74]; *p* < 2 × 10^−16^). Dose of the *APOE* ε4 allele was associated with a strong deleterious effect on the hazard of AD (discovery HR: 2.50 [95% CI: 2.43–2.57], *p* < 2 × 10^−16^; replication HR: 2.50 [95% CI: 2.41–2.59], *p* < 2 × 10^−16^).

#### 3.3.2 GWAS sensitivity to covariates

We performed two additional GWASs to evaluate the sensitivity of our results to covariate adjustment. The first alternative GWAS mimicked our original GWAS but removed *APOE* covariates while still adjusting for sex, population structure, and relatedness (Figure S15, Tables S2 and S3 in supporting information). The second alternative GWAS further simplified the model by removing sex as a covariate while still adjusting for population structure and relatedness (Figure S16, Tables S4 and S5 in supporting information). Relative to the original GWAS, the two alternative GWASs identified similar numbers of loci reaching significance and shared similar evidence for genomic inflation, but our original model recognized the greatest number of significant loci. Nine loci failed to reach genome‐wide significance across all three meta‐analyses models: 5q33.3, 7p21.3, 7q34‐q35, 12q24.31, 16q23.2, 17p13.2, 17p11.2, 18p11.21, and 19q13.32 (Table S6 in supporting information). The change at 19q13.32, the *APOE* locus, is to be expected. Most other loci had only modest changes in effect size estimates, but many failed to reach a suggestive threshold in at least one analysis. The signals at 5q33.3, 7q34‐q35, 17p11.2, and 18p11.21 needed *APOE* covariate adjustment to reach significance, while the signal at 12q24.31 needed sex as a covariate. This suggests that *APOE* genotype and sex covariates act as precision variables in GWAS for AD‐related traits.

#### 3.3.3 Effect across the genome

**FIGURE 4:**
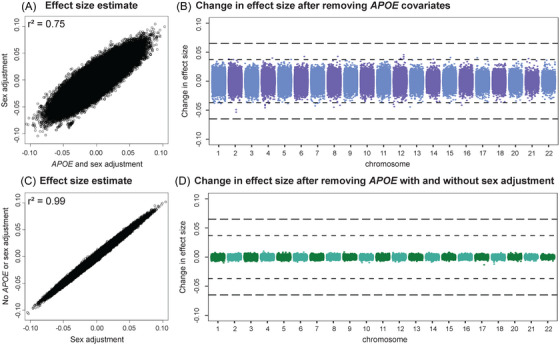
Change in effect size (β) estimates across GWAS models. Top: Contrast effect size estimates from the original GWAS with the first alternative GWAS adjusted for sex but not *APOE* in panel (A), and the difference between the two values in panel (B). Bottom: Contrast effect size estimates from the first alternative GWAS and the second that did not adjust for sex or *APOE* in panel (C), and the difference between the two in panel (D). All models adjust for population structure and relatedness. Results on chr19 are excluded to avoid *cis* effects due to *APOE* genotype. Dashed line: effect size comparable to the lead variant at 2q14.3 near *BIN1*. Dotted line: effect size comparable to the lead variant at 6p12.3 near *CD2AP*. *APOE*, apolipoprotein E; GWAS, genome‐wide association study.

Because we saw changes in both effect size and *p* value across models for some loci outside the *APOE* region, we explored the extent to which our covariate selection influenced our results across the genome. The inconsistent handling of covariates could possibly explain some of the variable support for previously reported AD GWAS SNPs at shared loci. A comparison of our original GWAS to the first alternative GWAS without *APOE* adjustment for all autosomes (excluding chromosome 19 where *APOE* is located) revealed correlated β estimates (*r* ^2^ = 0.75), which fluctuated by as much as the magnitude of a genome‐wide significant signal (Figure 4A, B). In contrast, comparison of the two alternative GWASs with and without sex adjustment revealed highly correlated β estimates (*r* ^2^ = 0.99) with fluctuations more comparable in magnitude to the standard errors at significant loci (Figure 4C, D). The stronger fluctuations due to *APOE* adjustment are likely explained by population structure, as the frequency distribution of *APOE* ε2 and ε4 are correlated with geography. In neither comparison do we see strong outliers.

### 3.4 Connections between GWAS signals and AD biology

**FIGURE 5:**
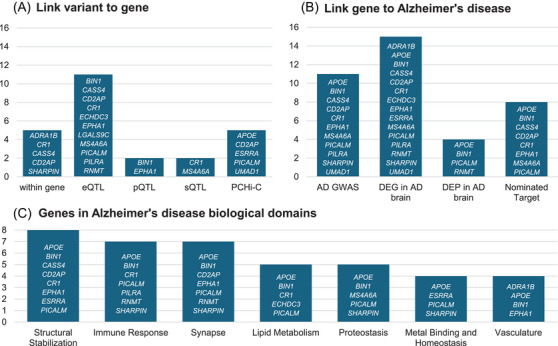
Connections between AAO GWAS hits and AD biology. Detailed annotations are provided in Table S7 in supporting information. A, Lead variants from the AAO GWAS are linked to genes based on their position (“within gene”) and their association with gene expression (“eQTL”), protein expression (“pQTL”), gene splicing (“sQTL”), and gene‐promoter interaction (promoter capture Hi‐C, “PC‐HiC”) as reported in Open Targets Genetics. B, Genes are linked to AD biology based on the strength of their association with AD by the Alzheimer's Disease Sequencing Project's Gene Verification Committee (“AD GWAS”), evidence the gene is differentially expressed (“DEG in AD brain”) or the protein is differentially expressed (“DEP in AD brain”) based on the Accelerating Medicines Partnership Program for Alzheimer's Disease consortium work, and their nomination as a therapeutic target as reported in Agora. C, Distribution of prioritized genes within biological domains linked to AD as reported in Agora, restricted to those supported by 4+ genes. AAO, age at onset; AD, Alzheimer's disease; GWAS, genome‐wide association study.

The lead variant at each original AAO GWAS signal (Table 2) was linked to a prioritized gene through its genetic and epigenetic context, then each prioritized gene was linked to AD biology through multi‐omics and systems biology (Table S7 in supporting information). Most variants (13/16, 81%) were linked to their prioritized gene through gene regulation, measured by direct association with transcript abundance (expression quantitative trait loci, eQTLs) or promoter‐gene interactions (promoter capture Hi‐C; Figure 5A). Similarly, nearly all prioritized genes were significantly differentially expressed in the AD brain (15/16, 94%; Figure 5B). Most prioritized genes (11/16, 69%) are robustly associated with late‐onset AD, while the 10p14 locus prioritizing *ECHDC3* has also long been associated with AD and family history of AD.^3^, ^4^, ^22^, ^36^, ^61^, ^62^, ^64^, ^65^, ^69^, ^70^ The heterogenous signals at 5q33.3 and 17p11.2 do not implicate the AD risk gene at the same locus (Table 3), while their implicated genes have relatively modest links to AD biology: *ADRA1B* at 5q33.3 is differentially expressed in the AD brain and belongs to the vasculature AD domain, while *LGALS9C* at 17p11.2 is not associated with gene or protein expression in the AD brain. In contrast, the two novel AAO GWAS signals at 11q13.1 and 18p11.21 implicate genes differentially expressed in the AD brain and involved in multiple AD biological domains: *ESRRA* is involved in structural stabilization, metal binding and homeostasis, and epigenetic AD domains, while *RNMT* is involved in the immune response and synapse AD domains. The prioritized genes represent most AD biological domains (16/19, 84%), excepting autophagy, DNA repair, and the RNA spliceosome. While structural stabilization, immune response, and synapse have the strongest support, 7/19 (37%) AD biological domains are implicated by at least a quarter of the prioritized genes (Figure 5C). Half of the prioritized genes have been independently nominated as AD therapeutic targets in Agora.

## 4 DISCUSSION

### 4.1 Overview

Despite a relatively modest sample size, our multi‐ancestry GWAS of a powerful survival‐based AAO of AD phenotype revealed new insights into known GWAS loci and nominated new genetic modifiers of AD. Variants at known AD risk loci were significantly associated with AAO of AD while analyzing ≈ 5% (5q33.3, 17p11.2, 8q24.3, 7p21.3) to 50% (20q13.31, 10p14, 7q22.1) the original AD GWAS sample size. We found that *APOE* adjustment exposed an independent signal on 19q13.32 and influenced variant effect sizes across the genome while sex adjustment did not. Despite the diversity within and between the discovery and replication data sets, our GWAS showed less evidence of polygenicity and genomic inflation than AD GWAS meta‐analyses restricted to non‐Hispanic Whites (smaller λ, comparable LD score regression intercepts)^3^, ^4^, ^62^, ^65^, ^66^, ^67^ while variant associations showed similar levels of heterogeneity.^57^

### 4.2 Shared genetic architecture with AD

Most of the AAO GWAS signals intersected AD risk loci (14/16, 88%). Three of these signals share the same lead variant as AD GWAS restricted to non‐Hispanic Whites (3/14, 21%; rs6733839 near *BIN1*, rs7920721 near *ECHDC3*, and rs34173062 in *SHARPIN*). This suggests these signals are transferable across populations and phenotypic measures. In contrast, six others lead variants are on the same haplotype (high D’) but with different allele frequencies (*r* ^2^ < 0.8) than their corresponding AD GWAS lead variant: 6p12.3, 7q22.1, 11q12.2, 11q14.2, 17p11.2, and 19q13.32. In half the cases, the AD GWAS SNP at these loci didn't reach genome‐wide significance, which suggests that fine mapping these loci in diverse populations might identify different drivers of association than those identified in strictly non‐Hispanic White samples. This multi‐ancestry AAO GWAS also uncovered independent signals at three AD GWAS loci: 5q33.3, 7q34‐q35, and 17p11.2. Together, this may help explain the attenuated portability of polygenic risk scores for AD across populations.

### 4.3 Novel loci associated with AAO of AD

This study identified two loci not previously associated with AD risk and variation at the APOE locus independently associated with AAO of AD. Preliminary research reveals that each novel locus implicates genes and biological processes with established links to AD. The AAO GWAS signal on 11q13.1 implicated ESRRA, the gene encoding estrogen‐related receptor alpha. Agora reports that ESRRA is differentially expressed in the AD brain and involved in the structural stabilization, metal binding and homeostasis, and epigenetic AD domains. ESRRA is also implicated in the pathogenesis of AD by studies of AD microglia, in vitro neuronal cells, and in vivo mouse models of AD.^71^, ^72^, ^73^ The AAO GWAS signal on 18p11.2 implicates *RNMT*, the gene encoding RNA guanine‐7 methyltransferase. Agora reports both the gene and protein are differentially expressed in AD and involved in both the immune response and synapse AD domains.^60^ RNMT plays an important role in mRNA modification with m7G, which has been implicated in aging and AD.^74^ Finally, the AAO GWAS signal on 19q33.3 independent of ε2 and ε4 genotypes is driven by rs73052335, an intronic *APOC1* variant associated with *APOE* regulation (Figure 5). This *APOC1* variant is most common in European and most rare in African reference populations and associated with an increased hazard of AD; this suggests it may explain some of the protective effect associated with African local ancestry at *APOE*.^33^, ^34^

### 4.4 *APOE* and sex effects on AAO of AD and GWAS signals

Both female sex and ε2 allele dosage were consistently associated with reduced hazard of AD, while ε4 dosage was associated with a strong and consistent increased hazard of AD. ε2 and ε4 genotypes acted as precision variables in this study, pulling suggestive signals up to significance and shining a light on SNPs within the shadow of *APOE*. The impact of including precision variables or non‐confounding covariates differs across regression strategies: while adjustment increases precision in linear regression, non‐confounding covariate adjustment can reduce power in logistic regression analyses of case/control status. We therefore recommend, when possible, researchers compare GWAS results from analyses with and without *APOE* adjustment. In this study, *APOE* covariate adjustment influenced effect size estimates across the genome. This is consistent with prior AD GWAS, which revealed significant associations that were sharply attenuated in a secondary analysis either adjusting for or stratifying by ε4.^22^, ^28^, ^69^ Rather than a biological interaction, this phenomenon is likely explained by the strong association between ε2 and ε4 with fine‐scale population structure in Europe and across the globe.^75^ In contrast, adjusting for sex as a covariate had minimal effect on variant effect sizes across the genome. This indicates that the observed changes in effect size with *APOE* adjustment are not driven by changes in the number of covariates alone.

### 4.5 Limitations and future directions

This study has several limitations. Our reliance on imputed data with strict QC eliminated a substantial number of variants (e.g., 19% in the discovery data harmonization). This prevented our study from investigating some known association signals, such as the *ABCA7* locus, which was significant in the discovery data but was missing too many genotypes to pass QC in the replication data. This missing data problem severely limits our ability to fine‐map the variant or variants driving the observed association signals. Our meta‐analysis strategy relied on the RE2 model to allow for heterogeneity expected when analyzing imputed markers and diverse data sets, but it can lead to association signals with opposing directions of effect across studies. Age at diagnosis is difficult to precisely measure, and age at last evaluation was not always captured in older data sets. Further analysis of sequence‐level variation in large, diverse data sets ascertained for AD are necessary to determine whether these meta‐analysis signals represent true disease associations rather than background noise. Together, these limitations underscore the need for large, diverse cohorts such as the Alzheimer's Disease Sequencing Project (ADSP) and All of Us, which pair genome sequence and robust phenotypic data to adequately power discovery and validation efforts.^20^

## CONFLICT OF INTEREST STATEMENT

The authors report no disclosures relevant to the manuscript. Author disclosures are available in the supporting information.

## Supporting information



Supporting information

Supporting information

Supporting information

## ADGC Collaborators

### 1

Farid Rajabli^1,2^, Penelope Benchek^3^, Giuseppe Tosto^4,5^, Gyungah R. Jun^6,7,8^, Christiane Reitz^9,10,5^, Nicholas Kushch^2^, Jin Sha^11^, Katrina Bazemore^11^, Congcong Zhu^6^, Wan‐Ping Lee^12^, Jacob Haut^11^, Kara L. Hamilton‐Nelson^2^, Nicholas R. Wheeler^3,13^, Yi Zhao^12^, John J. Farrell^6^, Michelle A. Grunin^3^, Yuk Yee Leung^12^, Pavel P. Kuksa^12^, Donghe Li^6^, Eder Lucio da Fonseca^2^, Jesse B. Mez^14^, Ellen L. Palmer^3^, Jagan Pillai^15^, Richard M. Sherva^16^, Yeunjoo E. Song^13,3^, Xiaoling Zhang^6,7^, Takeshi Ikeuchi^17^, Taha Iqbal^11^, Omkar Pathak^12^, Otto Valladares^12^, Dolly Reyes‐Dumeyer^4,5^, Amanda B. Kuzma^12^, Erin Abner^18^, Larry D. Adams^1^, Perrie M. Adams^19^, Alyssa Aguirre^20^, Marilyn S. Albert^21^, Roger L. Albin^22,23,24^, Mariet Allen^25^, Lisa Alvarez^26^, Liana G. Apostolova^27,28^, Steven E. Arnold^29^, Sanjay Asthana^30,31,32^, Craig S. Atwood^30,31,32^, Stanford Auerbach^14^, Gayle Ayres^20^, Clinton T. Baldwin^6^, Robert C. Barber^26^, Lisa L. Barnes^33,34,35^, Sandra Barral^4,10,5^, Thomas G. Beach^36^, James T. Becker^37^, Gary W. Beecham^2^, Duane Beekly^38^, Bruno A. Benitez^39^, David Bennett^33,35^, John Bertelson^40^, Thomas D. Bird^41,42^, Deborah Blacker^43,44^, Bradley F. Boeve^45^, James D. Bowen^46^, Adam Boxer^47^, James Brewer^48^, James R. Burke^49^, Jeffrey M. Burns^50^, Joseph D. Buxbaum^51,52,53^, Nigel J. Cairns^54^, Laura B. Cantwell^12^, Chuanhai Cao^55^, Christopher S. Carlson^56^, Cynthia M. Carlsson^32,31^, Regina M. Carney^57^, Minerva M. Carrasquillo^25^, Scott Chasse^58^, Marie‐Francoise Chesselet^59^, Nathaniel A. Chin^30,31^, Helena C. Chui^60^, Jaeyoon Chung^6^, Suzanne Craft^61^, Paul K. Crane^62^, David H. Cribbs^63^, Elizabeth A. Crocco^64^, Carlos Cruchaga^65,66^, Michael L. Cuccaro^2,1^, Munro Cullum^19^, Eveleen Darby^67^, Barbara Davis^68^, Philip L. De Jager^69^, Charles DeCarli^70^, John DeToledo^71^, Malcolm Dick^72^, Dennis W. Dickson^25^, Beth A. Dombroski^12^, Rachelle S. Doody^67^, Ranjan Duara^73^, NIlüfer Ertekin‐Taner^25,74^, Denis A. Evans^75^, Kelley M. Faber^76^, Thomas J. Fairchild^77^, Kenneth B. Fallon^78^, David W. Fardo^79^, Martin R. Farlow^80^, Victoria Fernandez‐Hernandez^39^, Steven Ferris^81^, Robert P. Friedland^82^, Tatiana M. Foroud^76^, Matthew P. Frosch^83^, Brian Fulton‐Howard^84^, Douglas R. Galasko^48^, Adriana Gamboa^85,86^, Marla Gearing^87,88^, Daniel H. Geschwind^59^, Bernardino Ghetti^89^, John R. Gilbert^2,1^, Rodney C.P. Go^78^, Alison M. Goate^51^, Thomas J. Grabowski^42,90^, Neill R. Graff‐Radford^25,74^, Robert C. Green^91^, John H. Growdon^92^, Hakon Hakonarson^93,94^, James Hall^26^, Ronald L. Hamilton^95^, Oscar Harari^66^, John Hardy^96,97^, Lindy E. Harrell^98^, Elizabeth Head^99^, Victor W. Henderson^100,101^, Michelle Hernandez^71^, Timothy Hohman^102,103^, Lawrence S. Honig^4^, Ryan M. Huebinger^104^, Matthew J. Huentelman^105^, Christine M. Hulette^106^, Bradley T. Hyman^92^, Linda S. Hynan^19,107,108^, Laura Ibanez^109,110^, Gail P. Jarvik^111,112^, Suman Jayadev^42^, Lee‐Way Jin^113^, Kim Johnson^71^, Leigh Johnson^85^, M. Ilyas Kamboh^114,115,116^, Anna M. Karydas^47^, Mindy J. Katz^117^, John S. Kauwe^118,119^, Jeffrey A. Kaye^120,121^, C. Dirk Keene^122^, Aisha Khaleeq^67^, Masataka Kikuchi^17^, Ronald Kim^99^, Janice Knebl^85^, Neil W. Kowall^14,123^, Joel H. Kramer^124^, Walter A. Kukull^125^, Frank M. LaFerla^126^, James J. Lah^127^, Eric B. Larson^128^, Alan Lerner^3^, James B. Leverenz^15^, Allan I. Levey^127^, Andrew P. Lieberman^129^, Richard B. Lipton^117^, Mark Logue^6,130,131^, Oscar L. Lopez^37^, Kathryn L. Lunetta^7^, Constantine G. Lyketsos^132^, Douglas Mains^85,86^, Flanagan E. Margaret^133,134^, Daniel C. Marson^98^, Eden R R. Martin^2,1^, Frank Martiniuk^135^, Deborah C. Mash^136^, Eliezer Masliah^48,137^, Paul Massman^67^, Arjun Masurkar^81^, Wayne C. McCormick^62^, Susan M. McCurry^138^, Andrew N. McDavid^56^, Stefan McDonough^139^, Ann C. McKee^14,140^, Marsel Mesulam^133,134^, Bruce L. Miller^141^, Carol A. Miller^142^, Joshua W. Miller^113^, Thomas J. Montine^143^, Edwin S. Monuki^144^, John C. Morris^54,145,146,110^, Shubhabrata Mukherjee^62^, Amanda J. Myers^64^, Trung Nguyen^107^, Obisesan Thomas Thomas Obisesan ADGC^147^, Sid O'Bryant^148^, John M. Olichney^149^, Marcia Ory^150^, Raymond Palmer^151^, Joseph E. Parisi^152^, Henry L. Paulson^22,24^, Valory Pavlik^67^, David Paydarfar^20^, Victoria Perez^71^, Elaine Peskind^153^, Ronald C. Petersen^45^, Helen Petrovitch^154^, Aimee Pierce^63^, Marsha Polk^151^, Wayne W. Poon^72^, Huntington Potter^155^, Liming Qu^12^, Mary Quiceno^156,157^, Joseph F. Quinn^120,121^, Ashok Raj^55^, Murray Raskind^153^, Eric M. Reiman^105,158,159,160^, Barry Reisberg^161,81^, Joan S. Reisch^68^, John M. Ringman^162^, Erik D. Roberson^98^, Monica Rodriguear^67^, Ekaterina Rogaeva^163^, Howard J. Rosen^47^, Roger N. Rosenberg^107^, Donald R. Royall^164^, Marwan Sabbagh^165^, A. Dessa Sadovnick^166^, Mark A. Sager^31^, Mary Sano^53^, Andrew J. Saykin^76,167^, Julie A. Schneider^33,35,168^, Lon S. Schneider^169,60^, William W. Seeley^47^, Susan H. Slifer^2^, Scott Small^4,5^, Amanda G. Smith^55^, Janet P. Smith^68^, Joshua A. Sonnen^122^, Salvatore Spina^89^, Peter St George‐Hyslop^170,171^, Takiyah D. Starks^172,173^, Robert A. Stern^14^, Alan B. Stevens^174,175,176^, Stephen M. Strittmatter^177^, David Sultzer^178^, Russell H. Swerdlow^50^, Rudolph E. Tanzi^92^, Jeffrey L. Tilson^179^, John Q. Trojanowski^12^, Juan C. Troncoso^180^, Magda Tsolaki^181^, Debby W. Tsuang^41,153^, Vivianna M. Van Deerlin^12^, Linda J. van Eldik^182^, Jeffery M. Vance^1,2^, Badri N. Vardarajan^4^, Robert Vassar^133,134^, Harry V. Vinters^183,162^, Jean‐Paul Vonsattel^4^, Sandra Weintraub^184^, Kathleen A. Welsh‐Bohmer^49,185^, Patrice L. Whitehead^2^, Kirk C. Wilhelmsen^58^, Benjamin Williams^186^, Jennifer Williamson^4^, Henrik Wilms^71^, Thomas S. Wingo^127^, Thomas Wisniewski^187,188^, Randall L. Woltjer^189^, Martin Woon^40^, Clinton B. Wright^190^, Chuang‐Kuo Wu^71^, Steven G. Younkin^25,74^, Chang‐En Yu^62^, Lei Yu^33,35^, Xiongwei Zhu^191^, Brian W. Kunkle^1,2^, William S. Bush^3,13^, Miyashita Akinori^17^, Byrd Goldie^192^, Li‐San Wang^12^, Lindsay A. Farrer^16,14,8,193,7^, Jonathan L. Haines^3,13^, Richard Mayeux^4^, Margaret A. Pericak‐Vance^1,2^, Gerard D. Schellenberg^12^

^1^Dr. John T. Macdonald Foundation Department of Human Genetics, Miller School of Medicine, University of Miami, Miami, Florida, USA; ^2^The John P. Hussman Institute for Human Genomics, University of Miami, Miami, Florida, USA; ^3^Department of Population and Quantitative Health Sciences, Case Western Reserve University, Cleveland, Ohio, USA; ^4^Taub Institute for Research in Alzheimer's Disease and the Aging Brain, The Gertrude H. Sergievsky Center, Department of Neurology, Columbia University, New York, New York, USA; ^5^Department of Neurology, Columbia University, New York, New York, USA; ^6^Department of Medicine (Biomedical Genetics), Boston University Chobanian & Avedisian School of Medicine, Boston, Massachusetts, USA; ^7^Department of Biostatistics, Boston University School of Public Health, Boston, Massachusetts, USA; ^8^Department of Ophthalmology, Boston University Chobanian & Avedisian School of Medicine, Boston, Massachusetts, USA; ^9^Department of Epidemiology, Columbia University, New York, New York, USA; ^10^Gertrude H. Sergievsky Center, Columbia University, New York, New York, USA; ^11^Department of Biostatistics, Epidemiology, and Informatics, Perelman School of Medicine, University of Pennsylvania, Philadelphia, Pennsylvania, USA; ^12^Penn Neurodegeneration Genomics Center, Department of Pathology and Laboratory Medicine, Perelman School of Medicine, University of Pennsylvania, Philadelphia, Pennsylvania, USA; ^13^Cleveland Institute for Computational Biology, Case Western Reserve University, Cleveland, Ohio, USA; ^14^Department of Neurology, Boston University Chobanian & Avedisian School of Medicine, Boston, Massachusetts, USA; ^15^Cleveland Clinic Lou Ruvo Center for Brain Health, Cleveland Clinic, Cleveland, Ohio, USA; ^16^Department of Medicine (Biomedical Genetics), Boston University Chobanian & Avedisian School of Medicine, Boston, Massachusetts, USA; ^17^Molecular Genetics Division, Brain Research Institute, Niigata University, Niigata, Japan; ^18^Sanders‐Brown Center on Aging, Department of Epidemiology, College of Public Health, University of Kentucky, Lexington, Kentucky, USA; ^19^Department of Psychiatry, University of Texas Southwestern Medical Center, Dallas, Texas, USA; ^20^Department of Neurology, Dell Medical School, University of Texas at Austin, Austin, Texas, USA; ^21^Department of Neurology, Johns Hopkins University, Baltimore, Maryland, USA; ^22^Department of Neurology, University of Michigan, Ann Arbor, Michigan, USA; ^23^Geriatric Research, Education and Clinical Center (GRECC), VA Ann Arbor Healthcare System (VAAAHS), Ann Arbor, Michigan, USA; ^24^Michigan Alzheimer's Disease Center, University of Michigan, Ann Arbor, Michigan, USA; ^25^Department of Neuroscience, Mayo Clinic, Jacksonville, Florida, USA; ^26^Department of Pharmacology and Neuroscience, University of North Texas Health Science Center, Fort Worth, Texas, USA; ^27^Departments of Neurology, Radiology, and Medical and Molecular Genetics, Indiana University School of Medicine, Indianapolis, Indiana, USA; ^28^Indiana Alzheimer's Disease Research Center, Indiana University School of Medicine, Indianapolis, Indiana, USA; ^29^Department of Psychiatry, Perelman School of Medicine, University of Pennsylvania, Philadelphia, Pennsylvania, USA; ^30^Geriatric Research, Education and Clinical Center (GRECC), University of Wisconsin, Madison, Wisconsin, USA; ^31^Department of Medicine, University of Wisconsin, Madison, Wisconsin, USA; ^32^Wisconsin Alzheimer's Disease Research Center, Madison, Wisconsin, USA; ^33^Department of Neurological Sciences, Rush University Medical Center, Chicago, Illinois, USA; ^34^Department of Behavioral Sciences, Rush University Medical Center, Chicago, Illinois, USA; ^35^Rush Alzheimer's Disease Center, Rush University Medical Center, Chicago, Illinois, USA; ^36^Civin Laboratory for Neuropathology, Banner Sun Health Research Institute, Phoenix, Arizona, USA; ^37^Departments of Psychiatry, Neurology, and Psychology, University of Pittsburgh School of Medicine, Pittsburgh, Pennsylvania, USA; ^38^National Alzheimer's Coordinating Center, University of Washington, Seattle, Washington, USA; ^39^Department of Psychiatry and Hope Center Program on Protein Aggregation and Neurodegeneration, Washington University School of Medicine, St. Louis, Missouri, USA; ^40^Department of Psychiatry, University of Texas at Austin/Dell Medical School, Austin, Texas, USA; ^41^VA Puget Sound Health Care System/GRECC, Seattle, Washington, USA; ^42^Department of Neurology, University of Washington, Seattle, Washington, USA; ^43^Department of Epidemiology, Harvard School of Public Health, Boston, Massachusetts, USA; ^44^Department of Psychiatry, Massachusetts General Hospital/Harvard Medical School, Boston, Massachusetts, USA; ^45^Department of Neurology, Mayo Clinic, Rochester, Minnesota, USA; ^46^Swedish Medical Center, Seattle, Washington, USA; ^47^Department of Neurology, University of California San Francisco, San Francisco, California, USA; ^48^Department of Neurosciences, University of California San Diego, La Jolla, California, USA; ^49^Department of Medicine, Duke University, Durham, North Carolina, USA; ^50^University of Kansas Alzheimer's Disease Center, University of Kansas Medical Center, Kansas City, Kansas, USA; ^51^Department of Genetics and Genomic Sciences, Ronald M. Loeb Center for Alzheimer's Disease, Icahn School of Medicine at Mount Sinai, New York, New York, USA; ^52^Department of Neuroscience, Icahn School of Medicine at Mount Sinai, New York, New York, USA; ^53^Department of Psychiatry, Mount Sinai School of Medicine, New York, New York, USA; ^54^Department of Pathology and Immunology, Washington University, St. Louis, Missouri, USA; ^55^USF Health Byrd Alzheimer's Institute, University of South Florida, Tampa, Florida, USA; ^56^Fred Hutchinson Cancer Research Center, Seattle, Washington, USA; ^57^Mental Health and Behavioral Science Service, Bruce W. Carter VA Medical Center, Miami, Florida, USA; ^58^Department of Genetics, University of North Carolina Chapel Hill, Chapel Hill, North Carolina, USA; ^59^Neurogenetics Program, University of California Los Angeles, Los Angeles, California, USA; ^60^Department of Neurology, University of Southern California, Los Angeles, California, USA; ^61^Section of Gerontology and Geriatric Medicine Research, Wake Forest School of Medicine, Winston‐Salem, North Carolina, USA; ^62^Department of Medicine, University of Washington, Seattle, Washington, USA; ^63^Department of Neurology, University of California Irvine, Irvine, California, USA; ^64^Department of Psychiatry and Behavioral Sciences, Miller School of Medicine, University of Miami, Miami, Florida, USA; ^65^NeuroGenomics and Informatics, Washington University, St. Louis, Missouri, USA; ^66^Department of Psychiatry, Washington University in St. Louis, St. Louis, Missouri, USA; ^67^Alzheimer's Disease and Memory Disorders Center, Baylor College of Medicine, Houston, Texas, USA; ^68^Department of Population and Data Sciences, University of Texas Southwestern Medical Center, Dallas, Texas, USA; ^69^Center for Translational and Computational Neuroimmunology, Department of Neurology, Columbia University Medical Center, New York, New York, USA; ^70^Department of Neurology, University of California Davis, Sacramento, California, USA; ^71^Departments of Neurology, Pharmacology and Neuroscience, Texas Tech University Health Science Center, Lubbock, Texas, USA; ^72^Institute for Memory Impairments and Neurological Disorders, University of California Irvine, Irvine, California, USA; ^73^Wien Center for Alzheimer's Disease and Memory Disorders, Mount Sinai Medical Center, Miami Beach, Florida, USA; ^74^Department of Neurology, Mayo Clinic, Jacksonville, Florida, USA; ^75^Rush Institute for Healthy Aging, Department of Internal Medicine, Rush University Medical Center, Chicago, Illinois, USA; ^76^Department of Medical and Molecular Genetics, Indiana University, Indianapolis, Indiana, USA; ^77^Office of Strategy and Measurement, University of North Texas Health Science Center, Fort Worth, Texas, USA; ^78^Department of Pathology, University of Alabama at Birmingham, Birmingham, Alabama, USA; ^79^Sanders‐Brown Center on Aging, Department of Biostatistics, College of Public Health, University of Kentucky, Lexington, Kentucky, USA; ^80^Department of Neurology, Indiana University, Indianapolis, Indiana, USA; ^81^Department of Psychiatry, New York University, New York, New York, USA; ^82^Department of Neurology, University of Louisville School of Medicine, Louisville, KY, USA; ^83^C.S. Kubik Laboratory for Neuropathology, Massachusetts General Hospital, Charlestown, Massachusetts, USA; ^84^Department of Neuroscience, Ronald M. Loeb Center for Alzheimer's Disease, Icahn School of Medicine at Mount Sinai, New York, New York, USA; ^85^Department of Health Behavior and Health Systems, University of North Texas Health Science Center, Fort Worth, Texas, USA; ^86^Department of Health Management and Policy, School of Public Health, University of North Texas Health Science Center, Fort Worth, Texas, USA; ^87^Department of Pathology and Laboratory Medicine, Emory University, Atlanta, Georgia, USA; ^88^Emory Alzheimer's Disease Center, Emory University, Atlanta, Georgia, USA; ^89^Department of Pathology and Laboratory Medicine, Indiana University, Indianapolis, Indiana, USA; ^90^Department of Radiology, University of Washington, Seattle, Washington, USA; ^91^Division of Genetics, Department of Medicine and Partners Center for Personalized Genetic Medicine, Brigham and Women's Hospital and Harvard Medical School, Boston, Massachusetts, USA; ^92^Department of Neurology, Massachusetts General Hospital/Harvard Medical School, Boston, Massachusetts, USA; ^93^Center for Applied Genomics, Children's Hospital of Philadelphia, Philadelphia, Pennsylvania, USA; ^94^Division of Human Genetics, Department of Pediatrics, Perelman School of Medicine, University of Pennsylvania, Philadelphia, Pennsylvania, USA; ^95^Department of Pathology (Neuropathology), University of Pittsburgh, Pittsburgh, Pennsylvania, USA; ^96^UCL Institute of Neurology, University College London, London, UK; ^97^Department of Molecular Neuroscience, UCL Institute of Neurology, University College London, London, UK; ^98^Department of Neurology, University of Alabama at Birmingham, Birmingham, Alabama, USA; ^99^Department of Pathology and Laboratory Medicine, University of California Irvine, Irvine, California, USA; ^100^Department of Epidemiology and Population Health, Stanford University, Stanford, California, USA; ^101^Department of Neurology and Neurological Sciences, Stanford University, Stanford, California, USA; ^102^Vanderbilt Memory and Alzheimer's Center, Department of Neurology, Vanderbilt University Medical Center, Nashville, Tennessee, USA; ^103^Vanderbilt Genetics Institute, Division of Genetic Medicine, Department of Medicine, Vanderbilt University Medical Center, Nashville, Tennessee, USA; ^104^Department of Surgery, University of Texas Southwestern Medical Center, Dallas, Texas, USA; ^105^Neurogenomics Division, Translational Genomics Research Institute, Phoenix, Arizona, USA; ^106^Department of Pathology, Duke University, Durham, North Carolina, USA; ^107^Department of Neurology, University of Texas Southwestern Medical Center, Dallas, Texas, USA; ^108^Department of Neurological Surgery, University of Texas Southwestern Medical Center, Dallas, Texas, USA; ^109^Department of Psychiatry, Washington University School of Medicine, St. Louis, Missouri, USA; ^110^Hope Center Program on Protein Aggregation and Neurodegeneration, Washington University School of Medicine, St. Louis, Missouri, USA; ^111^Department of Genome Sciences, University of Washington, Seattle, Washington, USA; ^112^Department of Medicine (Medical Genetics), University of Washington, Seattle, Washington, USA; ^113^Department of Pathology and Laboratory Medicine, University of California Davis, Sacramento, California, USA; ^114^Department of Psychiatry, University of Pittsburgh, Pittsburgh, Pennsylvania, USA; ^115^Department of Human Genetics, University of Pittsburgh, Pittsburgh, Pennsylvania, USA; ^116^Alzheimer's Disease Research Center, University of Pittsburgh, Pittsburgh, Pennsylvania, USA; ^117^Department of Neurology, Albert Einstein College of Medicine, New York, New York, USA; ^118^Department of Neuroscience, Brigham Young University, Provo, Utah, USA; ^119^Department of Biology, Brigham Young University, Provo, Utah, USA; ^120^Department of Neurology, Oregon Health and Science University, Portland, Oregon, USA; ^121^Department of Neurology, Portland Veterans Affairs Medical Center, Portland, Oregon, USA; ^122^Department of Laboratory Medicine and Pathology, University of Washington, Seattle, Washington, USA; ^123^Department of Pathology, Boston University, Boston, Massachusetts, USA; ^124^Department of Neuropsychology, University of California San Francisco, San Francisco, California, USA; ^125^Department of Epidemiology, University of Washington, Seattle, Washington, USA; ^126^Department of Neurobiology and Behavior, University of California Irvine, Irvine, California, USA; ^127^Department of Neurology, Emory University, Atlanta, Georgia, USA; ^128^Kaiser Permanente Washington Health Research Institute, Seattle, Washington, USA; ^129^Department of Pathology, University of Michigan, Ann Arbor, Michigan, USA; ^130^National Center for PTSD at Boston VA Healthcare System, Boston, Massachusetts, USA; ^131^Department of Psychiatry, Boston University Chobanian & Avedisian School of Medicine, Boston, Massachusetts, USA; ^132^Department of Psychiatry, Johns Hopkins University, Baltimore, Maryland, USA; ^133^Department of Pathology, Northwestern University Feinberg School of Medicine, Chicago, Illinois, USA; ^134^Cognitive Neurology and Alzheimer's Disease Center, Northwestern University Feinberg School of Medicine, Chicago, Illinois, USA; ^135^Department of Medicine ‐ Pulmonary, New York University, New York, New York, USA; ^136^Department of Neurology, Miller School of Medicine, University of Miami, Miami, Florida, USA; ^137^Department of Pathology, University of California San Diego, La Jolla, California, USA; ^138^School of Nursing Northwest Research Group on Aging, University of Washington, Seattle, Washington, USA; ^139^Pfizer Worldwide Research and Development, New York, New York, USA; ^140^Department of Pathology, Boston University Chobanian & Avedisian School of Medicine, Boston, Massachusetts, USA; ^141^Weill Institute for Neurosciences, Memory and Aging Center, University of California San Francisco, San Francisco, California, USA; ^142^Department of Pathology, University of Southern California, Los Angeles, California, USA; ^143^Department of Pathology, Stanford University School of Medicine, Stanford, California, USA; ^144^Department of Pathology and Laboratory Medicine and Alzheimer's Disease Research Center, University of California Irvine, Irvine, California, USA; ^145^Department of Neurology, Washington University, St. Louis, Missouri, USA; ^146^Department of Psychiatry, Washington University School of Medicine, St. Louis Missouri, USA; ^147^Department of Research Regulatory Compliance, College of Medicine, Howard University, Washington, DC, USA, tobisesan@Howard.edu; ^148^Institute for Translational Research, University of North Texas Health Science Center, Fort Worth, Texas, USA; ^149^Center for Mind and Brain and Department of Neurology, University of California Davis, Sacramento, California, USA; ^150^Center for Population Health and Aging, Texas A&M University Health Science Center, Lubbock Texas, USA; ^151^Department of Family and Community Medicine, University of Texas Health Science Center San Antonio, San Antonio, Texas, USA; ^152^Department of Laboratory Medicine and Pathology, Mayo Clinic, Rochester, Minnesota, USA; ^153^Department of Psychiatry and Behavioral Sciences, University of Washington School of Medicine, Seattle, Washington, USA; ^154^Pacific Health Research & Education Institute, VA Pacific Islands Healthcare System, Honolulu, Hawaii, USA; ^155^Department of Neurology, University of Colorado School of Medicine, Aurora, Colorado, USA; ^156^Department of Internal Medicine and Geriatrics, University of North Texas Health Science Center, Fort Worth, Texas, USA; ^157^Department of Medical Education, TCU/UNTHSC School of Medicine, Fort Worth, Texas;, ^158^Arizona Alzheimer's Consortium, Phoenix, Arizona, USA; ^159^Banner Alzheimer's Institute, Phoenix, Arizona, USA; ^160^Department of Psychiatry, University of Arizona, Phoenix, Arizona, USA; ^161^Alzheimer's Disease Center, New York University, New York, New York, USA; ^162^Department of Neurology, University of California Los Angeles, Los Angeles, California, USA; ^163^Tanz Centre for Research in Neurodegenerative Disease, University of Toronto, Toronto, Ontario, Canada; ^164^Departments of Psychiatry, Medicine, Family and Community Medicine, and the Glenn Biggs Institute for Alzheimer's and Neurodegenerative Diseases, UT Health Science Center at San Antonio, San Antonio, Texas, USA; ^165^Department of Neurology, Barrow Neurological Institute St. Joseph's Hospital and Medical Center, Phoenix, Arizona, USA; ^166^Department of Medical Genetics, University of British Columbia, Vancouver, Canada; ^167^Department of Radiology and Imaging Sciences, Indiana University, Indianapolis, Indiana, USA; ^168^Department of Pathology (Neuropathology), Rush University Medical Center, Chicago, Illinois, USA; ^169^Department of Psychiatry, University of Southern California, Los Angeles, California, USA; ^170^Cambridge Institute for Medical Research, University of Cambridge, Cambridge, UK; ^171^Faculty of Medicine, Department of Medicine (Neurology), University of Toronto, Toronto, Ontario, Canada; ^172^Maya Angelou Center for Health Equity, Wake Forest School of Medicine, Winston‐Salem, North Carolina, USA; ^173^Center for Outreach in Alzheimer's, Aging and Community Health at North Carolina A&T State University, Greensboro, North Carolina, USA; ^174^Center for Applied Health Research, Baylor Scott & White Health, Temple, Texas, USA; ^175^Center for Population Health and Aging, Texas A&M University Health Science Center, Lubbock Texas, USA; ^176^College of Medicine, Texas A&M University Health Science Center, College Station, Texas, USA; ^177^Program in Cellular Neuroscience, Neurodegeneration and Repair, Yale University School of Medicine, New Haven, Connecticut, USA;, ^178^Department of Psychiatry and Human Behavior, University of California Irvine, Irvine, California, USA; ^179^Renaissance Computing Institute, University of North Carolina Chapel Hill, Chapel Hill, North Carolina, USA; ^180^Department of Pathology, Johns Hopkins University, Baltimore, Maryland, USA; ^181^Department of Neurology, Aristotle University of Thessaloniki, Thessaloniki, Macedonia; ^182^Sanders‐Brown Center on Aging, Department of Neuroscience, College of Medicine, University of Kentucky, Lexington, Kentucky, USA; ^183^Department of Pathology and Laboratory Medicine, University of California Los Angeles, Los Angeles, California, USA; ^184^Department of Psychiatry and Behavioral Sciences, Northwestern University Feinberg School of Medicine, Chicago, Illinois, USA; ^185^Department of Psychiatry and Behavioral Sciences, Duke University, Durham, North Carolina, USA; ^186^Department of Neurology, Section of Gerontology and Geriatric Medicine Research, Wake Forest School of Medicine, Winston‐Salem, North Carolina, USA; ^187^Department of Psychiatry, New York University Grossman School of Medicine, New York, New York, USA; ^188^Center for Cognitive Neurology and Departments of Neurology and Pathology, New York University Grossman School of Medicine, New York, USA; ^189^Department of Pathology, Oregon Health and Science University, Portland, Oregon, USA; ^190^Evelyn F. McKnight Brain Institute, Department of Neurology, Miller School of Medicine, University of Miami, Miami, Florida, USA; ^191^Department of Pathology, Case Western Reserve University, Cleveland, Ohio, USA; ^192^Social Sciences & Health Policy, Wake Forest School of Medicine, Winston‐Salem, North Carolina, USA; ^193^Department of Epidemiology, Boston University School of Public Health, Boston, Massachusetts, USA.
