# Supplementary material for: Multi‐ancestry meta‐analysis identifies genetic modifiers of age‐at‐onset of Alzheimer's disease at known and novel loci
Source: Alzheimers Dement. 2025 Aug 29;21(9):e70489. doi: 10.1002/alz.70489 (PMC12397202; doi:10.1002/alz.70489)

Supplemental Materials

TITLE: Multi-ancestry meta-analysis identifies genetic modifiers of age-at-onset of Alzheimer's disease at known and novel loci

AUTHORS: Elizabeth E. Blue [1,2,3], Jai Broome[1,4], Diane Xue[2,5], Hanley Kingston[2,6], Nicola H. Chapman[1], Stephanie Gogarten[7], Alzheimer's Disease Genetics Consortium (ADGC), Adam C. Naj[8,9], Ellen M. Wijsman[1,7, 10]

[1]Department of Medicine, Division of Medical Genetics, University of Washington, Seattle, Washington, USA
[2]Institute for Public Health Genetics, University of Washington, Seattle, Washington, USA
[3]Brotman Baty Institute, University of Washington, Seattle, Washington, USA
[4]Department of Medicine, Division of General Internal Medicine, University of Washington, Seattle, Washington, USA
[5]Department of Genetics, Perelman School of Medicine, University of Pennsylvania, Philadelphia, Pennsylvania, USA
[6]San Mateo Public Health Laboratory, San Mateo, California, USA
[7]Department of Biostatistics, University of Washington, Seattle, Washington, USA
[8]Department of Biostatistics, Epidemiology, and Informatics, Perelman School of Medicine, University of Pennsylvania, Philadelphia, Pennsylvania, USA
[9]Penn Neurodegeneration Genomics Center, Department of Pathology and Laboratory Medicine, Perelman School of Medicine, University of Pennsylvania, Philadelphia, Pennsylvania, USA
[10]Department of Genome Sciences, University of Washington, Seattle, Washington, USA

*** CORRESPONDING AUTHOR**

Elizabeth E. Blue, Division of Medical Genetics, University of Washington, BOX 357720, Seattle, WA 98195-7720. Email: em27@uw.edu

Supplemental Figures

Supplemental Figure 1

Principal components (PC) analysis of the Discovery data.

A: Pairwise PC plots color coded by genotyping array. **B:** Pairwise PC plots color coded by reported race/ethnicity. **C:** Scree plot describing the percent variance explained by each of the first 32 PCs.

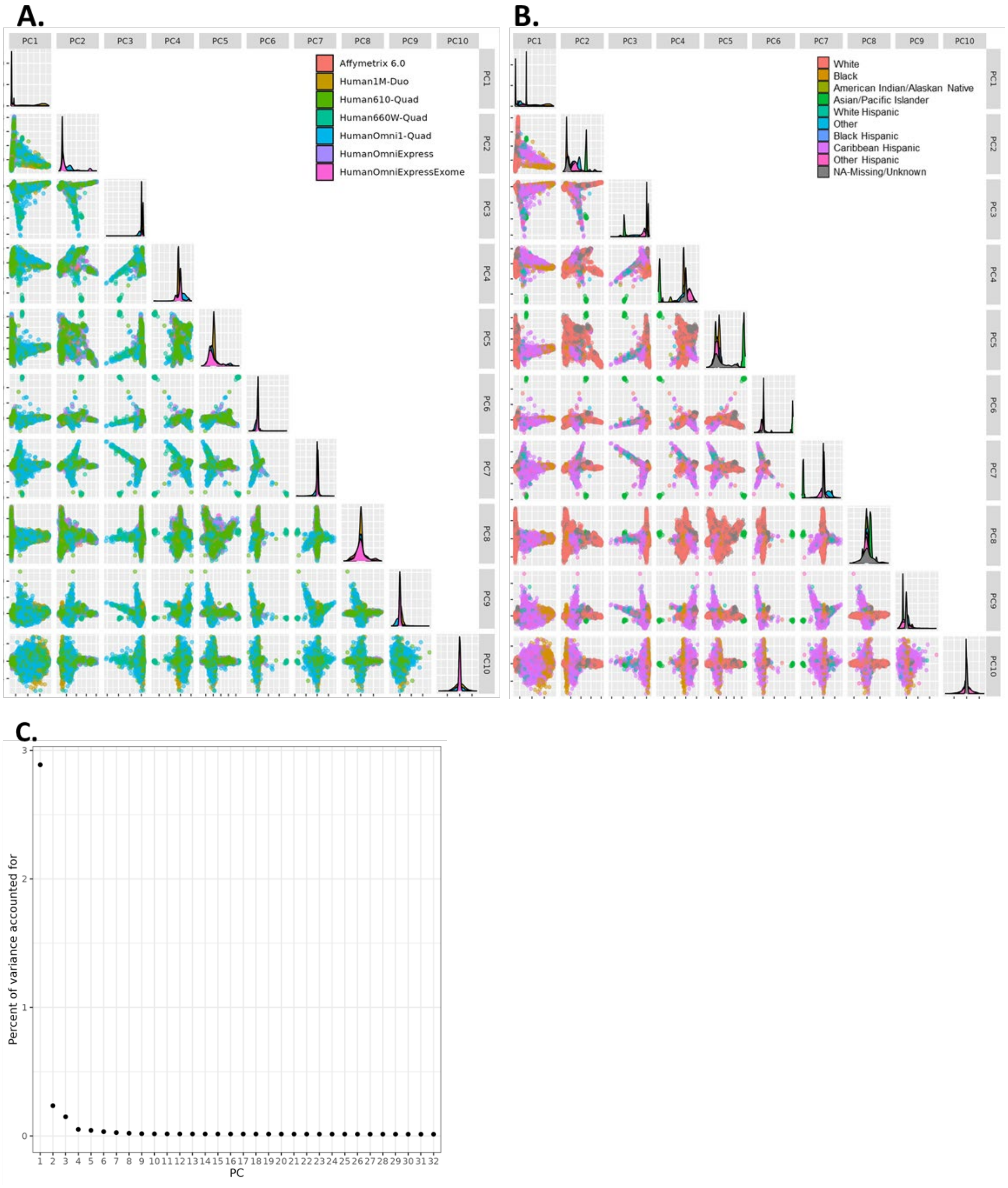

Supplemental Figure 2

Principal components (PC) analysis of the Replication data.

A: Pairwise PC plots color coded by genotyping array. **B:** Pairwise PC plots color coded by reported race/ethnicity. **C:** Scree plot describing the percent variance explained by each of the first 32 PCs.

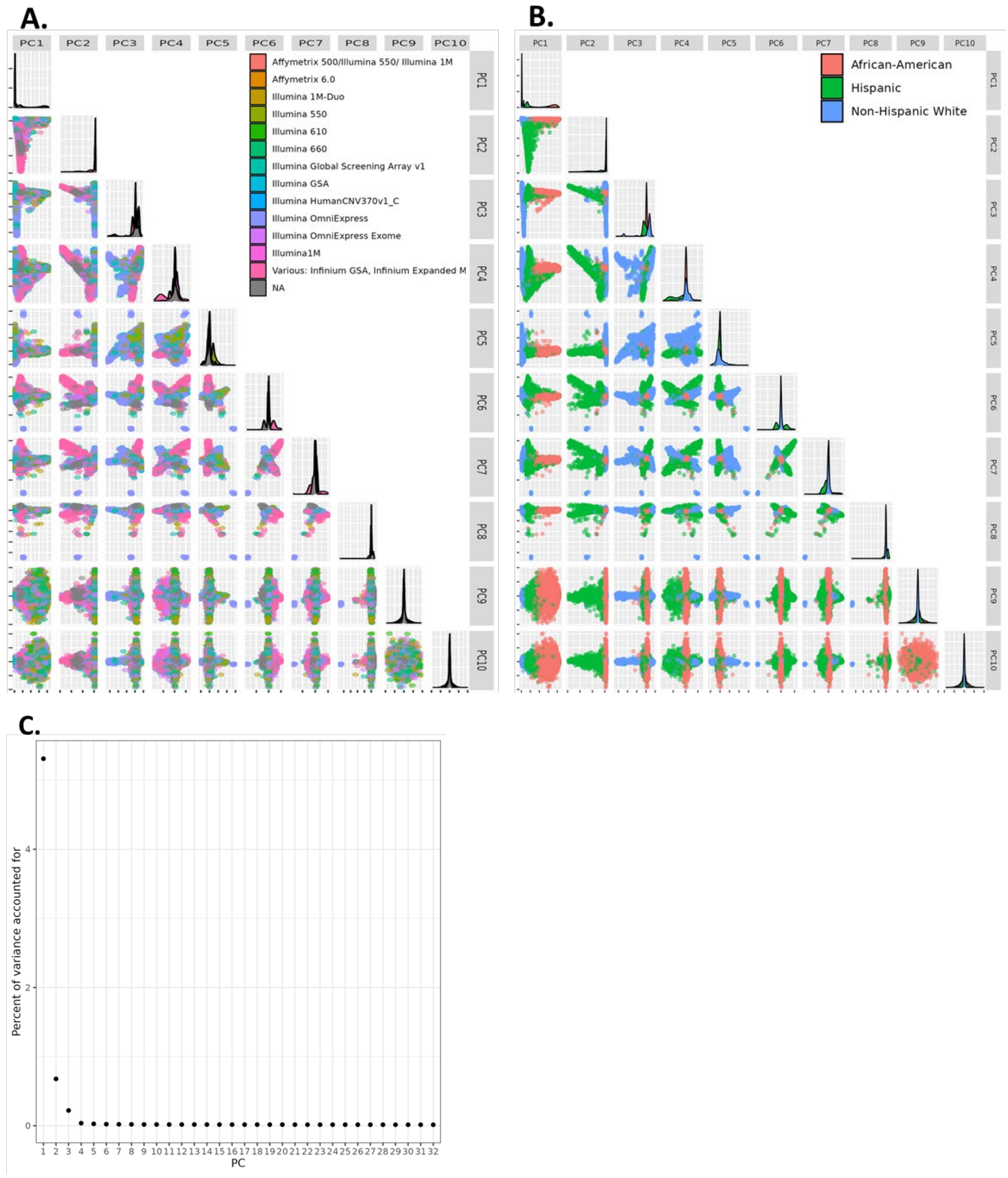

Supplemental Figure 3

LocusZoom plot for 2q14.3 near *BIN1*.

X-axis: Position on GRCh38. **Left Y-axis:** -log10P-values corresponding to data points. **Right Y-axis:** recombination rate, corresponding to the blue line. Points are color-coded with evidence for linkage disequilibrium (r^2) in the 1000 Genomes European reference sample. The candidate gene from prior GWAS is highlighted in red.

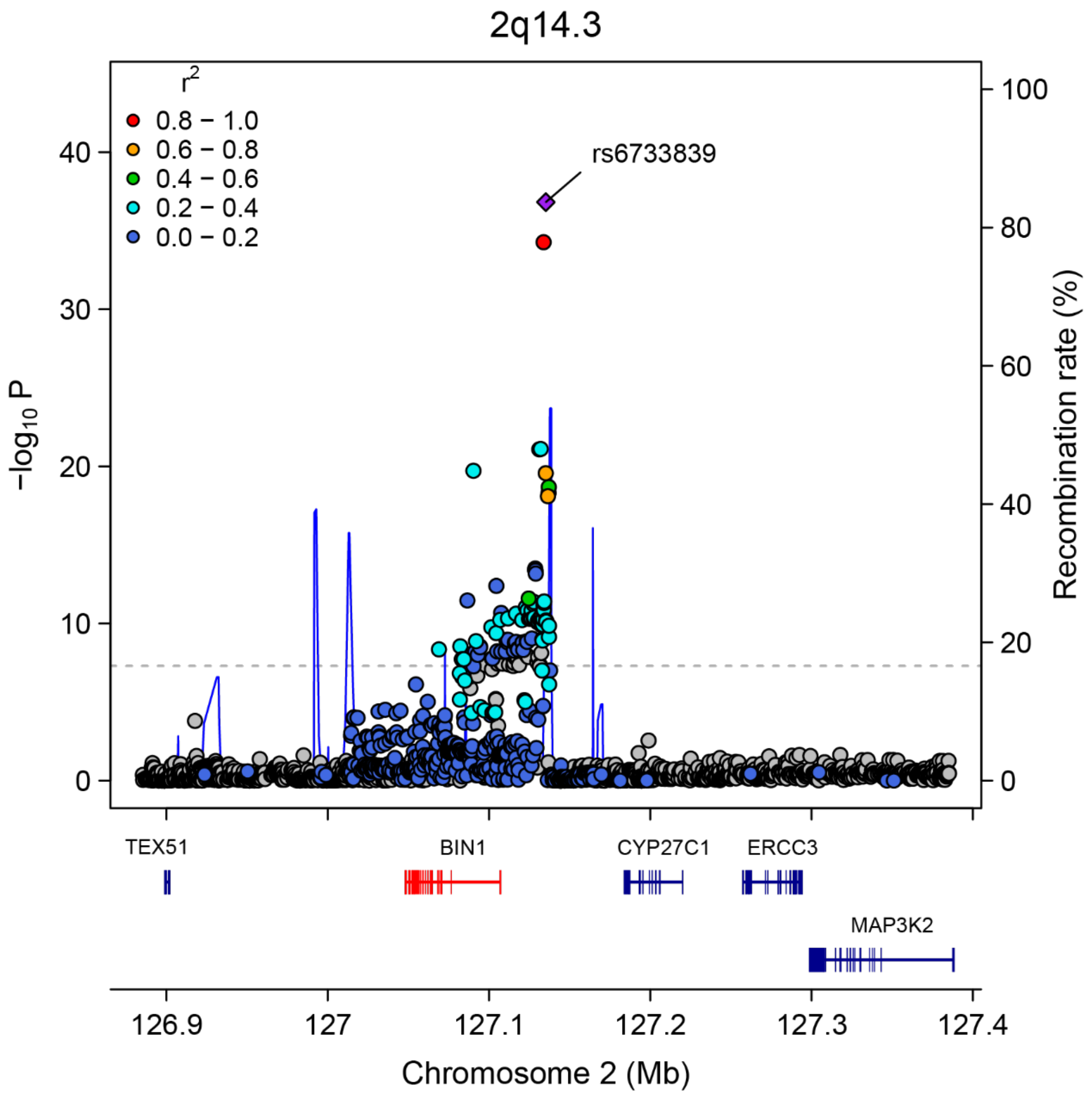

Supplemental Figure 4

LocusZoom for 10p14 near *ECHDC3.*

X-axis: Position on GRCh38. **Left Y-axis:** -log10P-values corresponding to data points. **Right Y-axis:** recombination rate, corresponding to the blue line. Points are color-coded with evidence for linkage disequilibrium (r^2) in the 1000 Genomes European reference sample. The candidate gene from prior GWAS is highlighted in red.

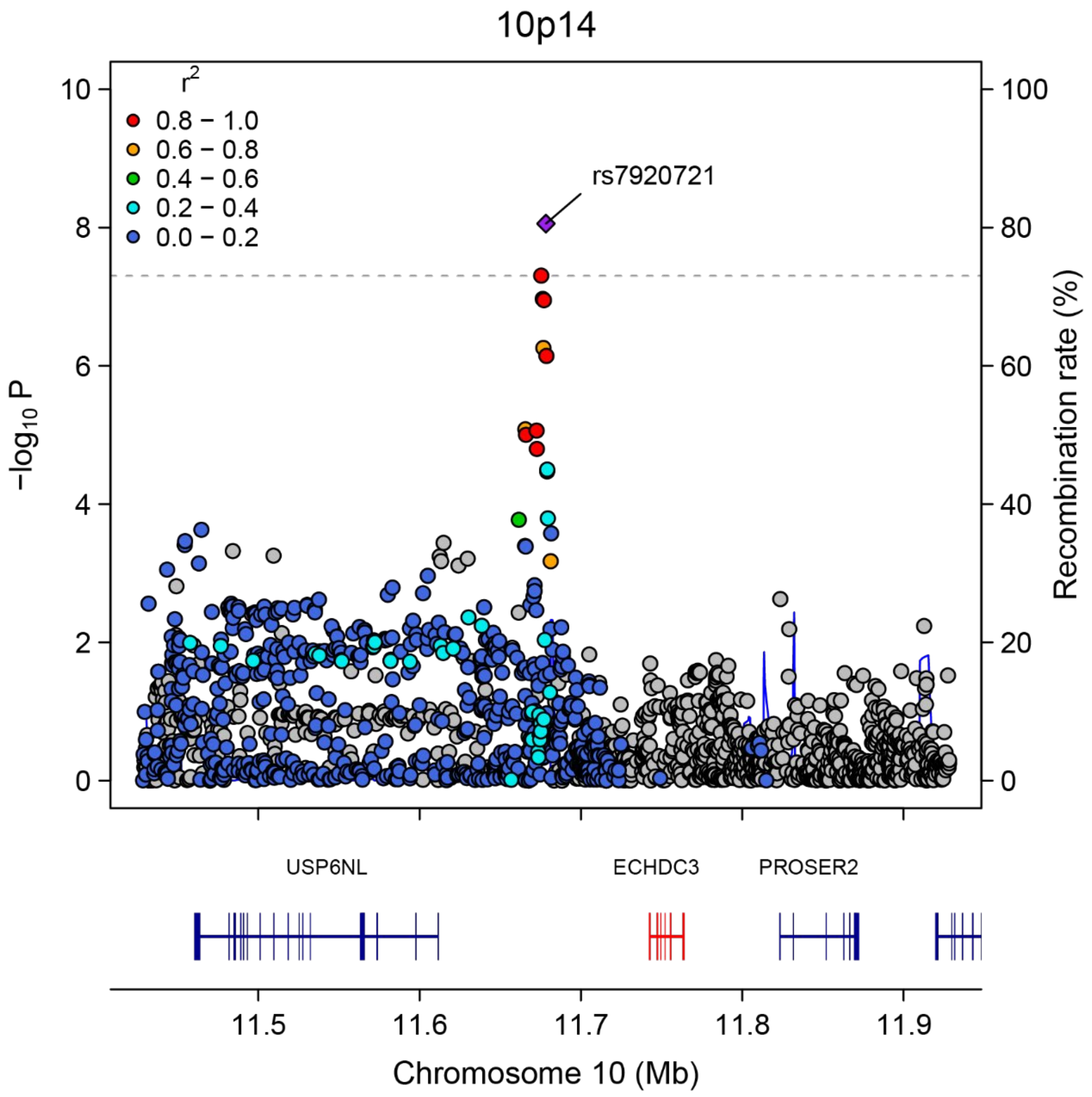

Supplemental Figure 5

LocusZoom for 8q24.3 near *SHARPIN.*

X-axis: Position on GRCh38. **Left Y-axis:** -log10P-values corresponding to data points. **Right Y-axis:** recombination rate, corresponding to the blue line. Points are color-coded with evidence for linkage disequilibrium (r^2) in the 1000 Genomes European reference sample. The candidate gene from prior GWAS is highlighted in red.

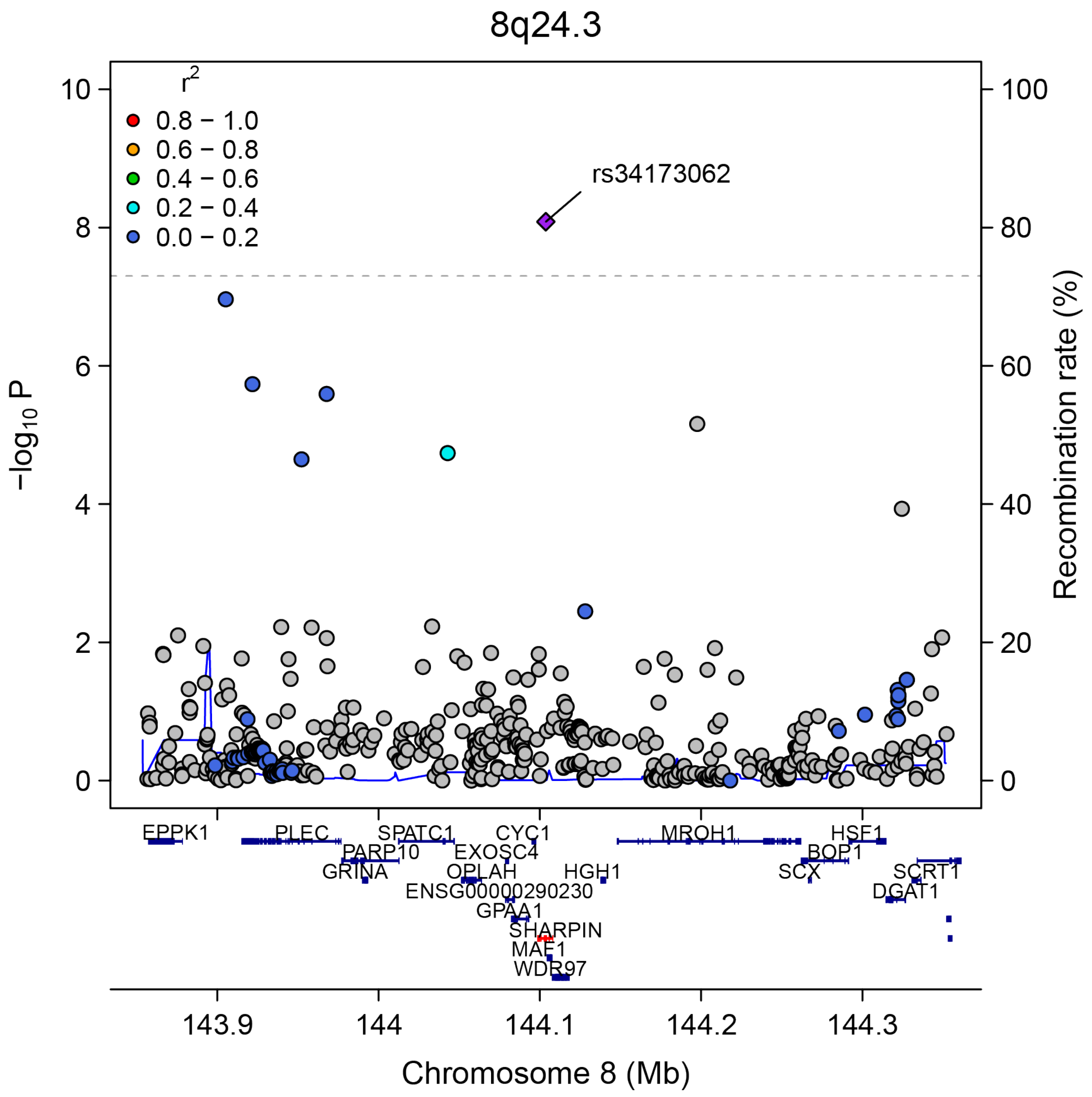

Supplemental Figure 6

LocusZoom for 1q32.2 near *CR1.*

X-axis: Position on GRCh38. **Left Y-axis:** -log10P-values corresponding to data points. **Right Y-axis:** recombination rate, corresponding to the blue line. Points are color-coded with evidence for linkage disequilibrium (r^2) in the 1000 Genomes European reference sample. The candidate gene from prior GWAS is highlighted in red.

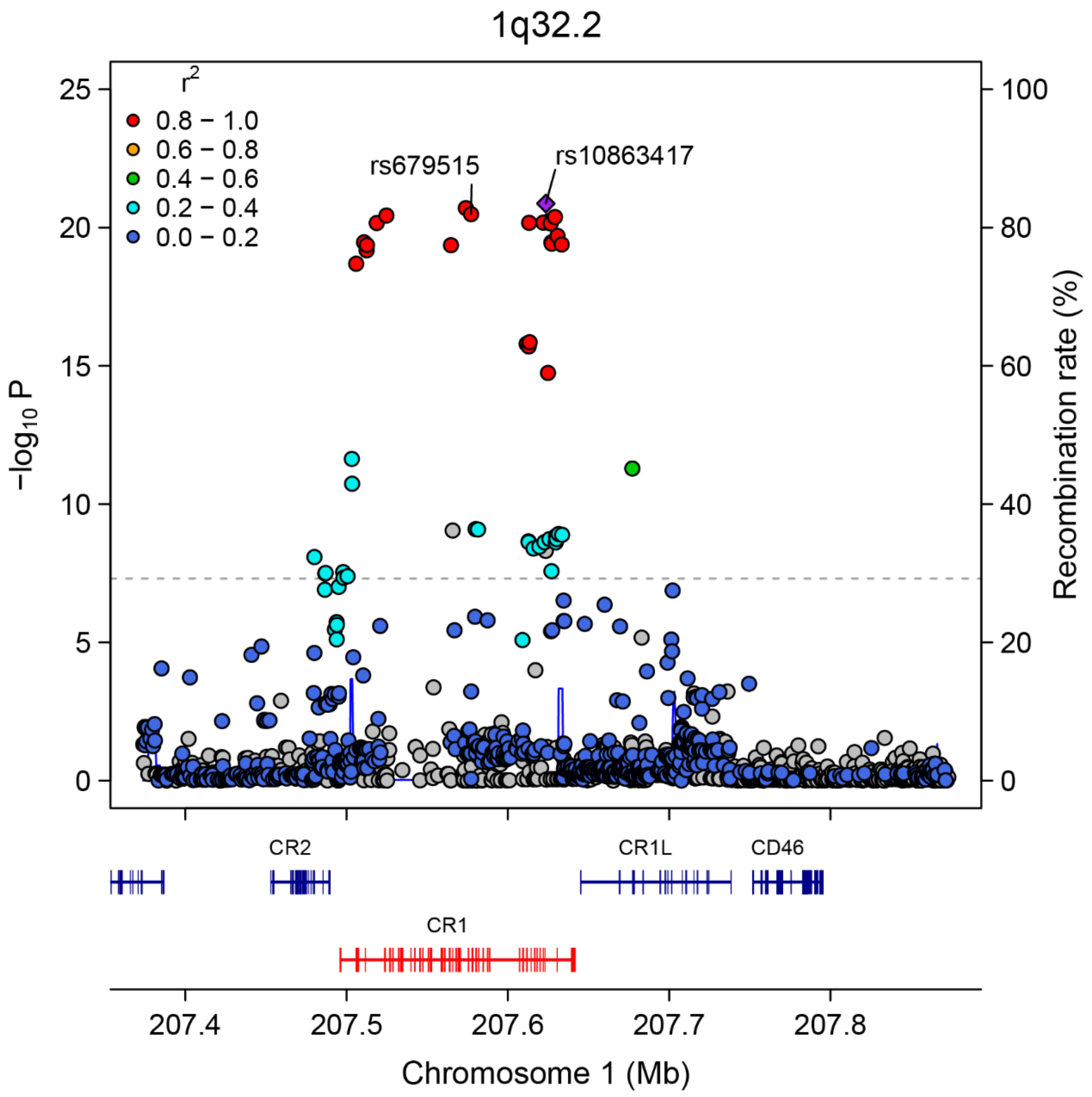

Supplemental Figure 7

LocusZoom for 7p21.3 near *UMAD1.*

X-axis: Position on GRCh38. **Left Y-axis:** -log10P-values corresponding to data points. **Right Y-axis:** recombination rate, corresponding to the blue line. Points are color-coded with evidence for linkage disequilibrium (r^2) in the 1000 Genomes European reference sample. The candidate gene from prior GWAS is highlighted in red.

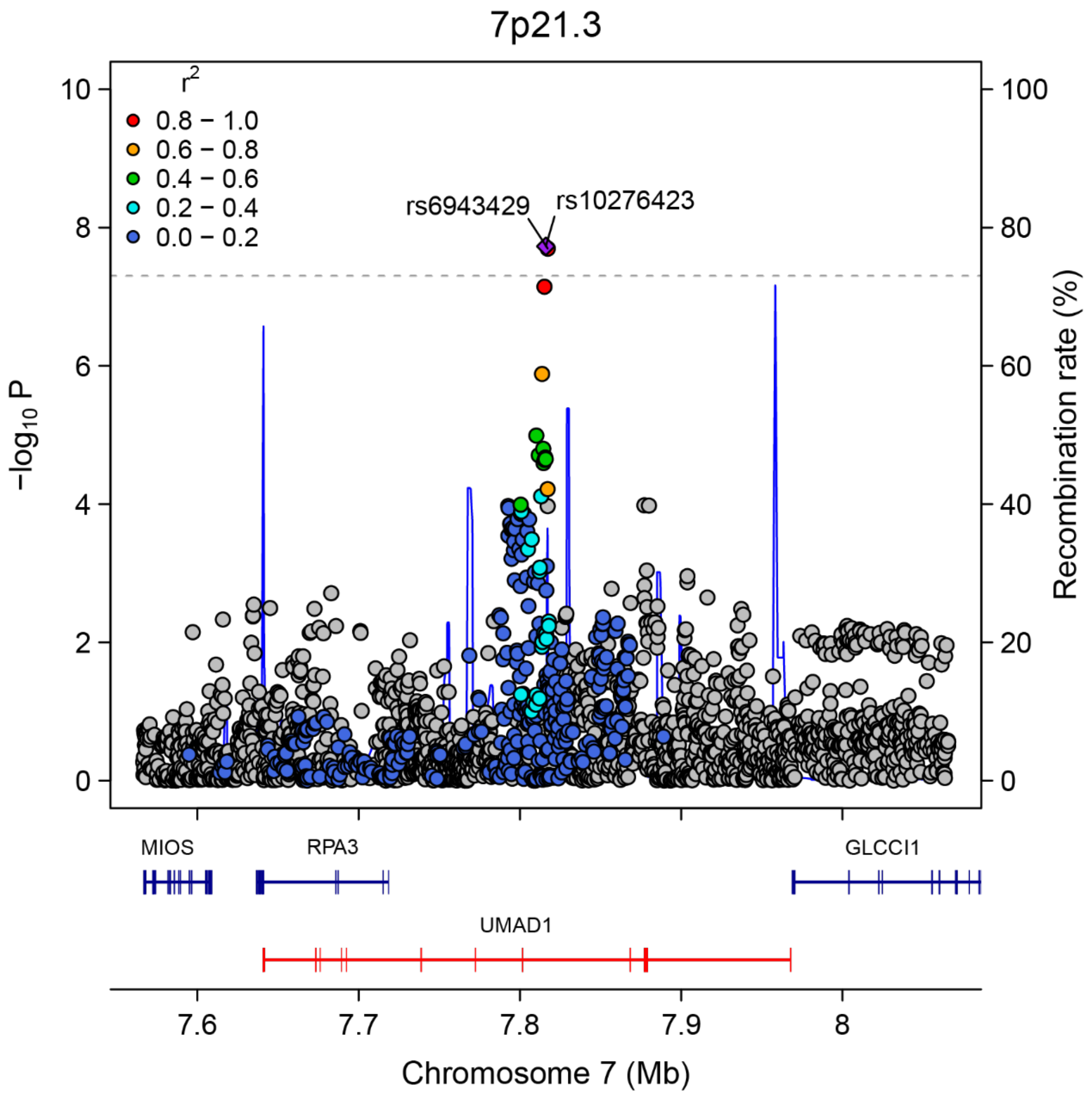

Supplemental Figure 8

LocusZoom for 11q12.2 near *MS4A6A*.

X-axis: Position on GRCh38. **Left Y-axis:** -log10P-values corresponding to data points. **Right Y-axis:** recombination rate, corresponding to the blue line. Points are color-coded with evidence for linkage disequilibrium (r^2) in the 1000 Genomes European reference sample. The candidate gene from prior GWAS is highlighted in red.

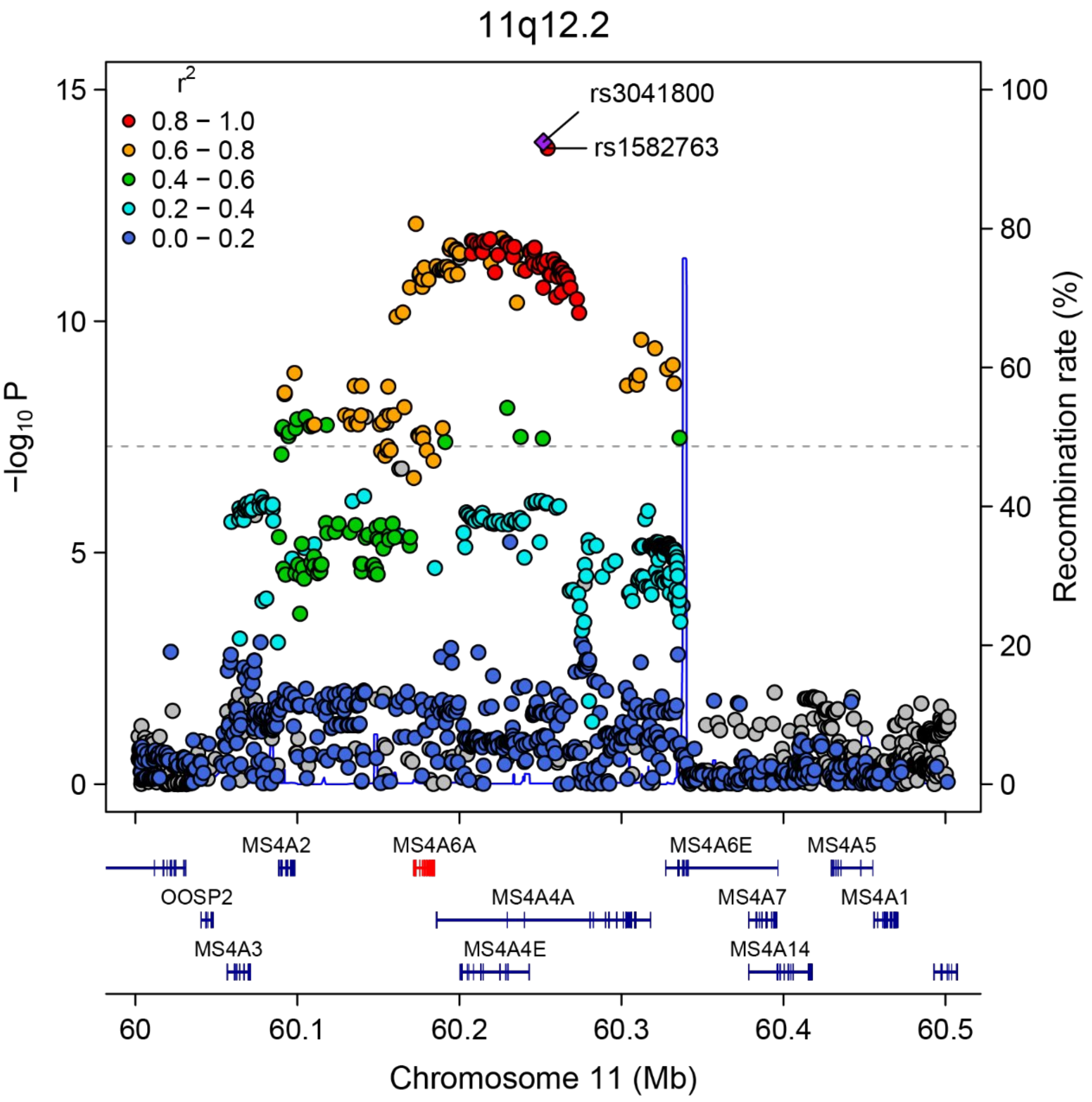

Supplemental Figure 9

LocusZoom for 6p12.13 near *CD2AP*.

X-axis: Position on GRCh38. **Left Y-axis:** -log10P-values corresponding to data points. **Right Y-axis:** recombination rate, corresponding to the blue line. Points are color-coded with evidence for linkage disequilibrium (r^2) in the 1000 Genomes European reference sample. The candidate gene from prior GWAS is highlighted in red.

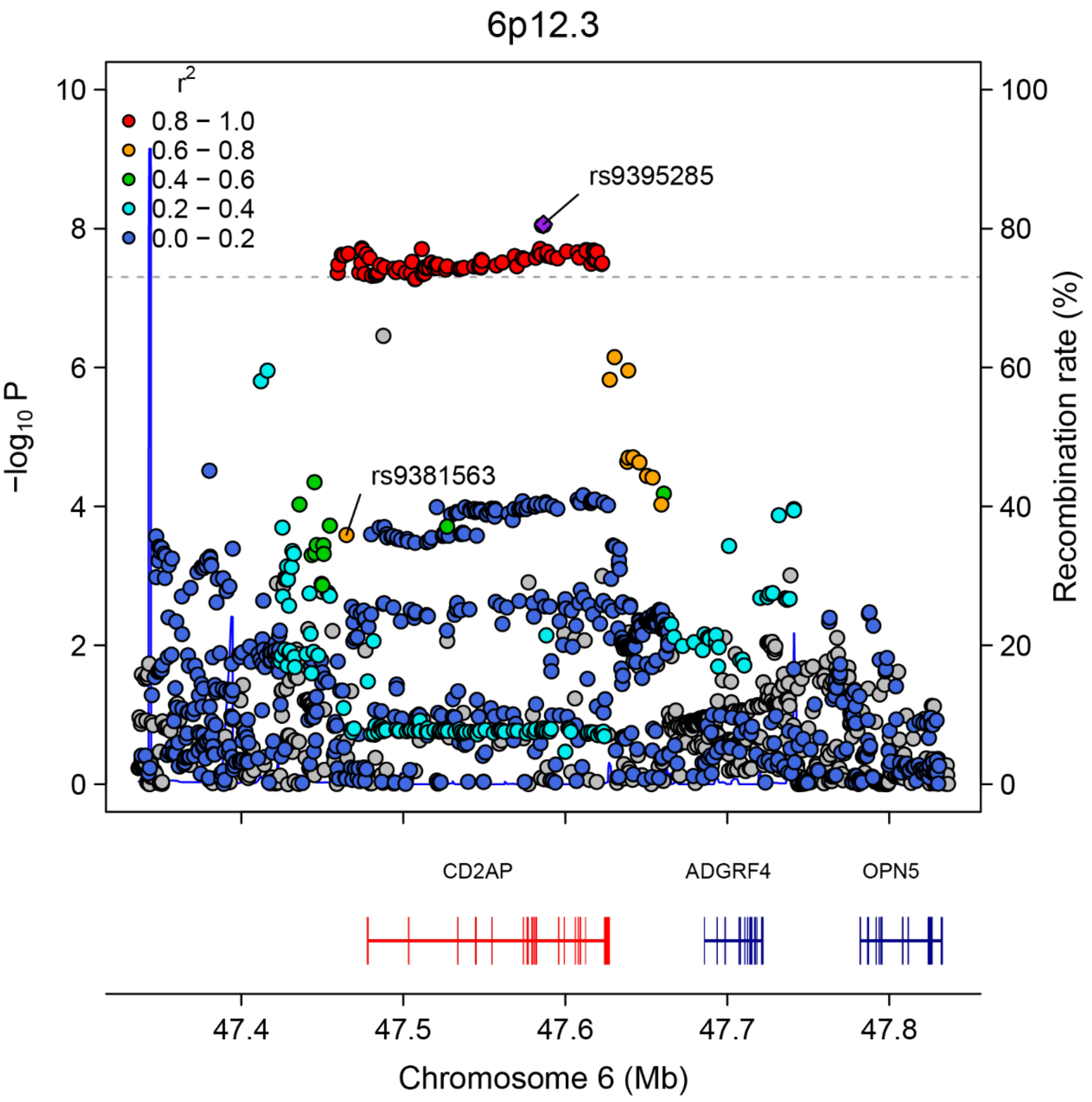

Supplemental Figure 10

LocusZoom for 7q22.1 near *PILRA.*

X-axis: Position on GRCh38. **Left Y-axis:** -log10P-values corresponding to data points. **Right Y-axis:** recombination rate, corresponding to the blue line. Points are color-coded with evidence for linkage disequilibrium (r^2) in the 1000 Genomes European reference sample. The candidate gene from prior GWAS is highlighted in red.

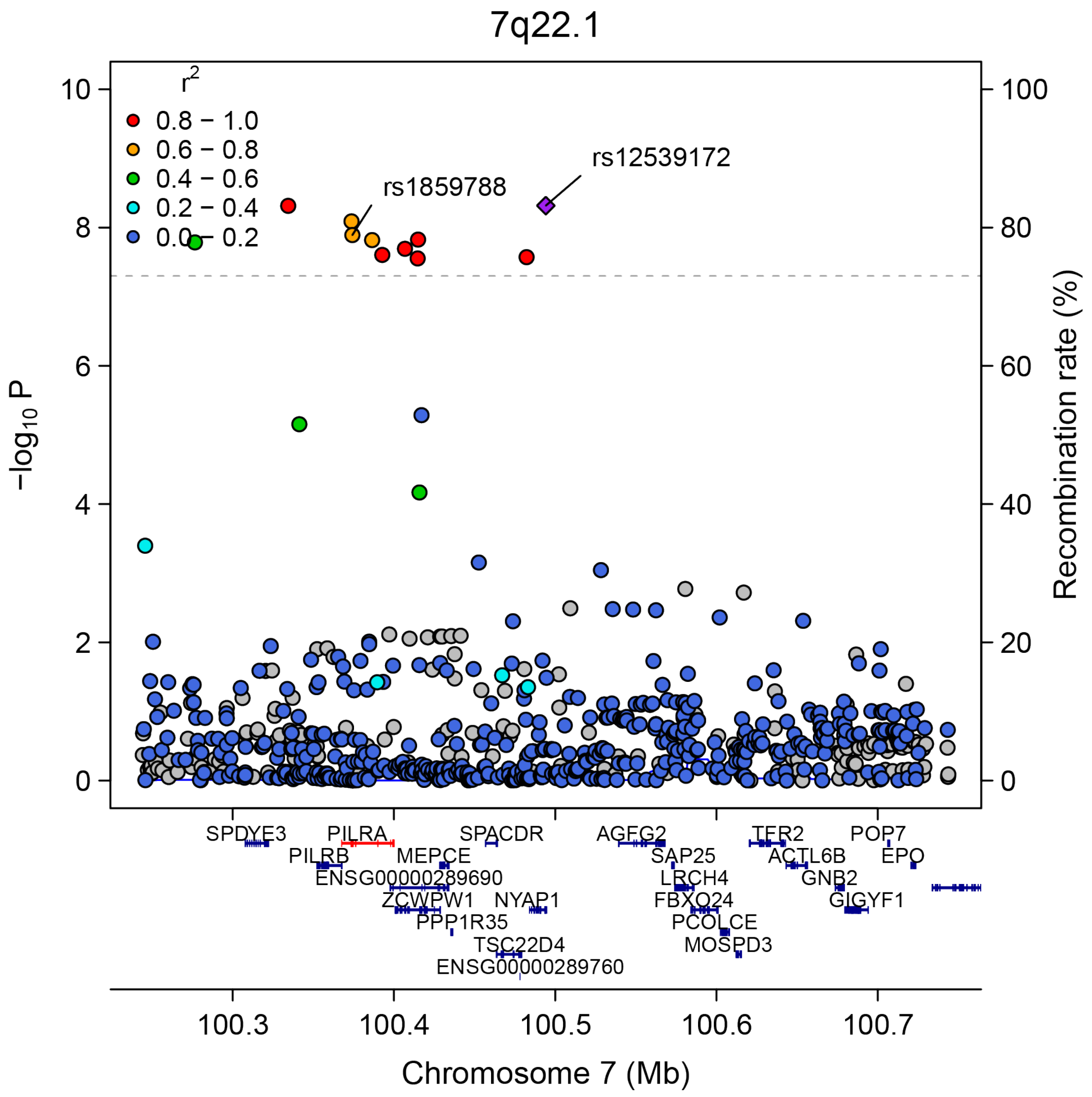

Supplemental Figure 11

LocusZoom for 11q14.2 near *PICALM*.

X-axis: Position on GRCh38. **Left Y-axis:** -log10P-values corresponding to data points. **Right Y-axis:** recombination rate, corresponding to the blue line. Points are color-coded with evidence for linkage disequilibrium (r^2) in the 1000 Genomes European reference sample. The candidate gene from prior GWAS is highlighted in red.

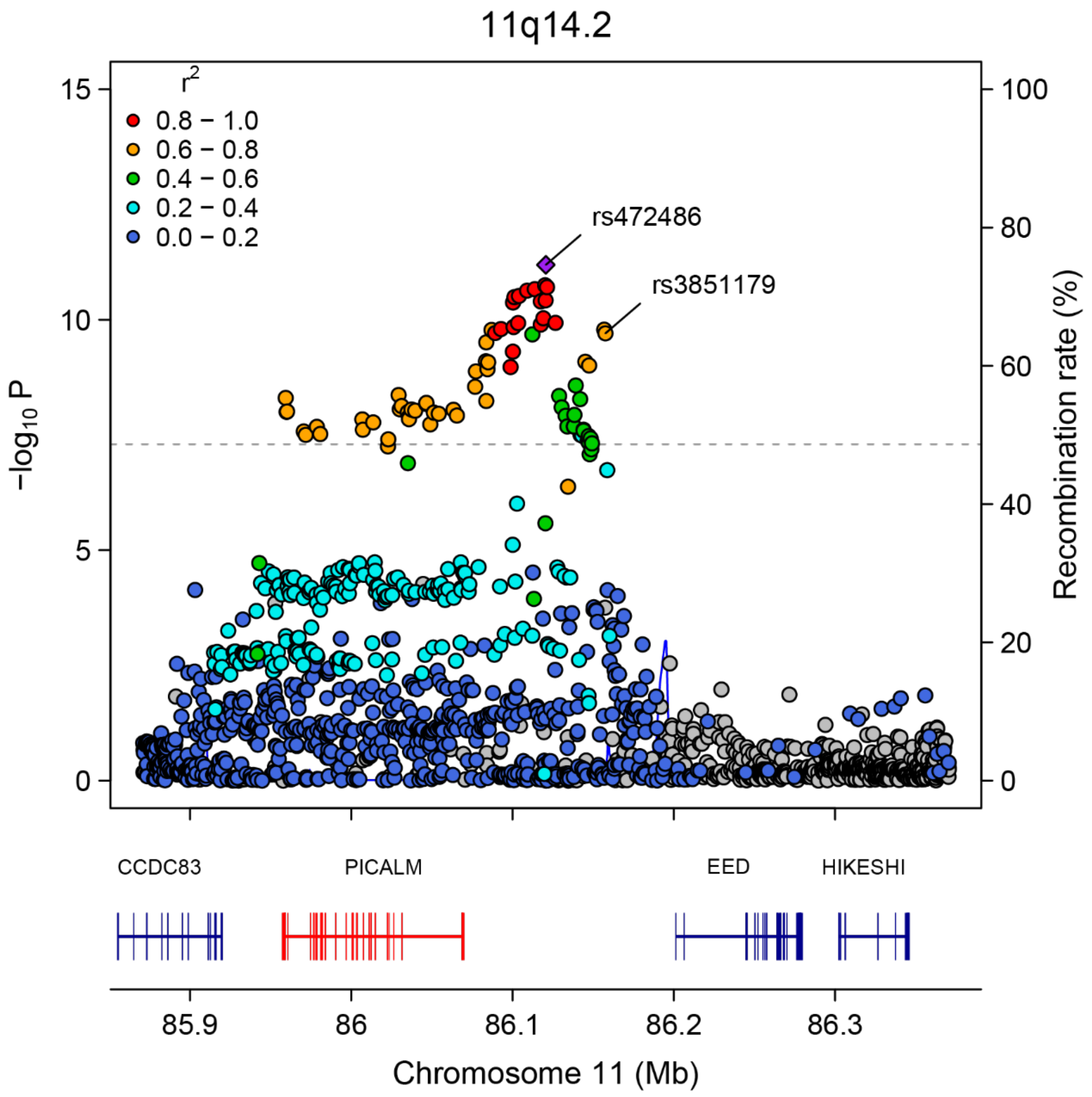

Supplemental Figure 12

LocusZoom for 20q13.31 near *CASS4*.

X-axis: Position on GRCh38. **Left Y-axis:** -log10P-values corresponding to data points. **Right Y-axis:** recombination rate, corresponding to the blue line. Points are color-coded with evidence for linkage disequilibrium (r^2) in the 1000 Genomes European reference sample. The candidate gene from prior GWAS is highlighted in red.

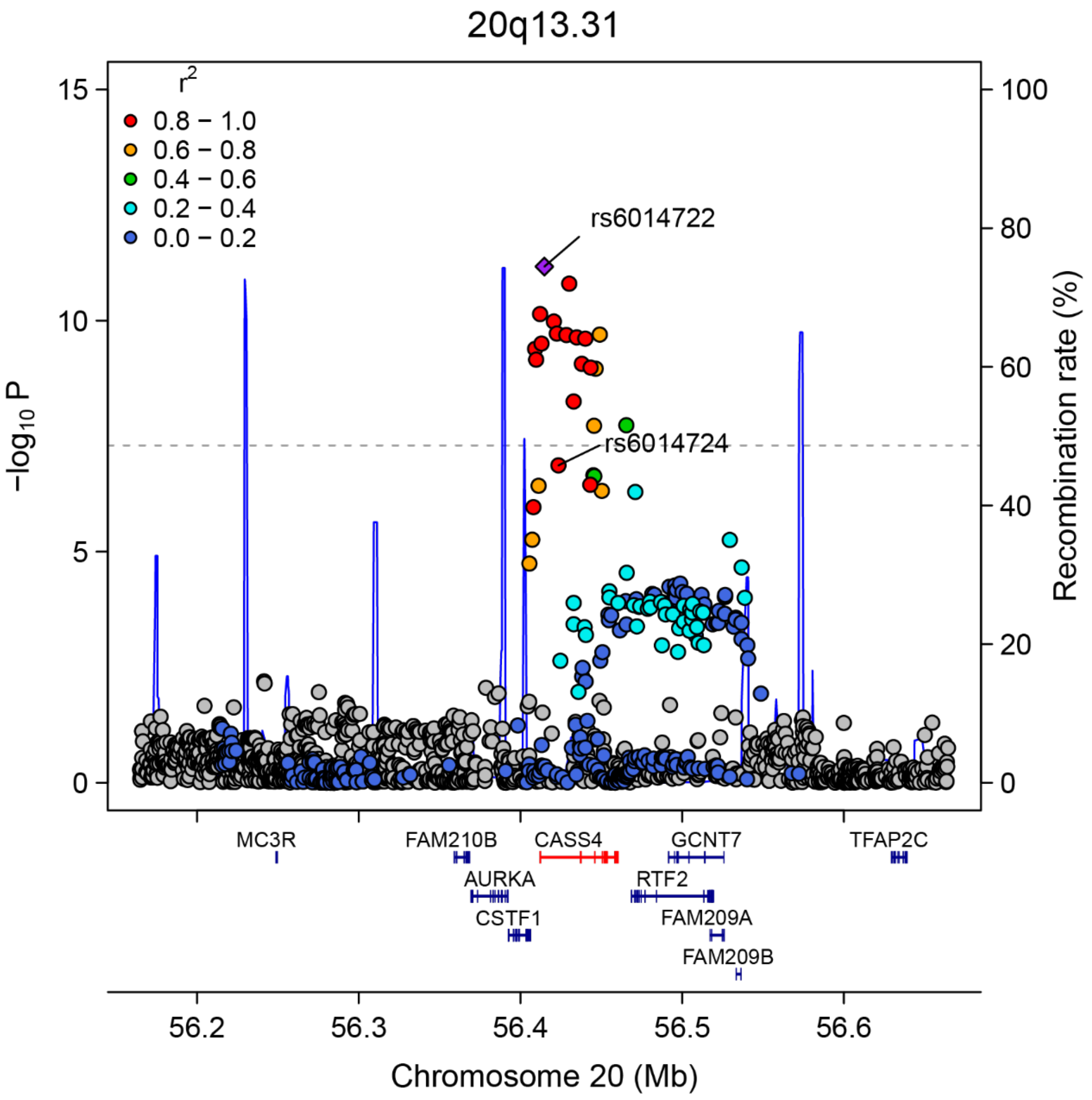

Supplemental Figure 13

LocusZoom for 7q34-q35 near *EPHA1*.

X-axis: Position on GRCh38. **Left Y-axis:** -log10P-values corresponding to data points. **Right Y-axis:** recombination rate, corresponding to the blue line. Points are color-coded with evidence for linkage disequilibrium (r^2) in the 1000 Genomes European reference sample. The candidate gene from prior GWAS is highlighted in red.

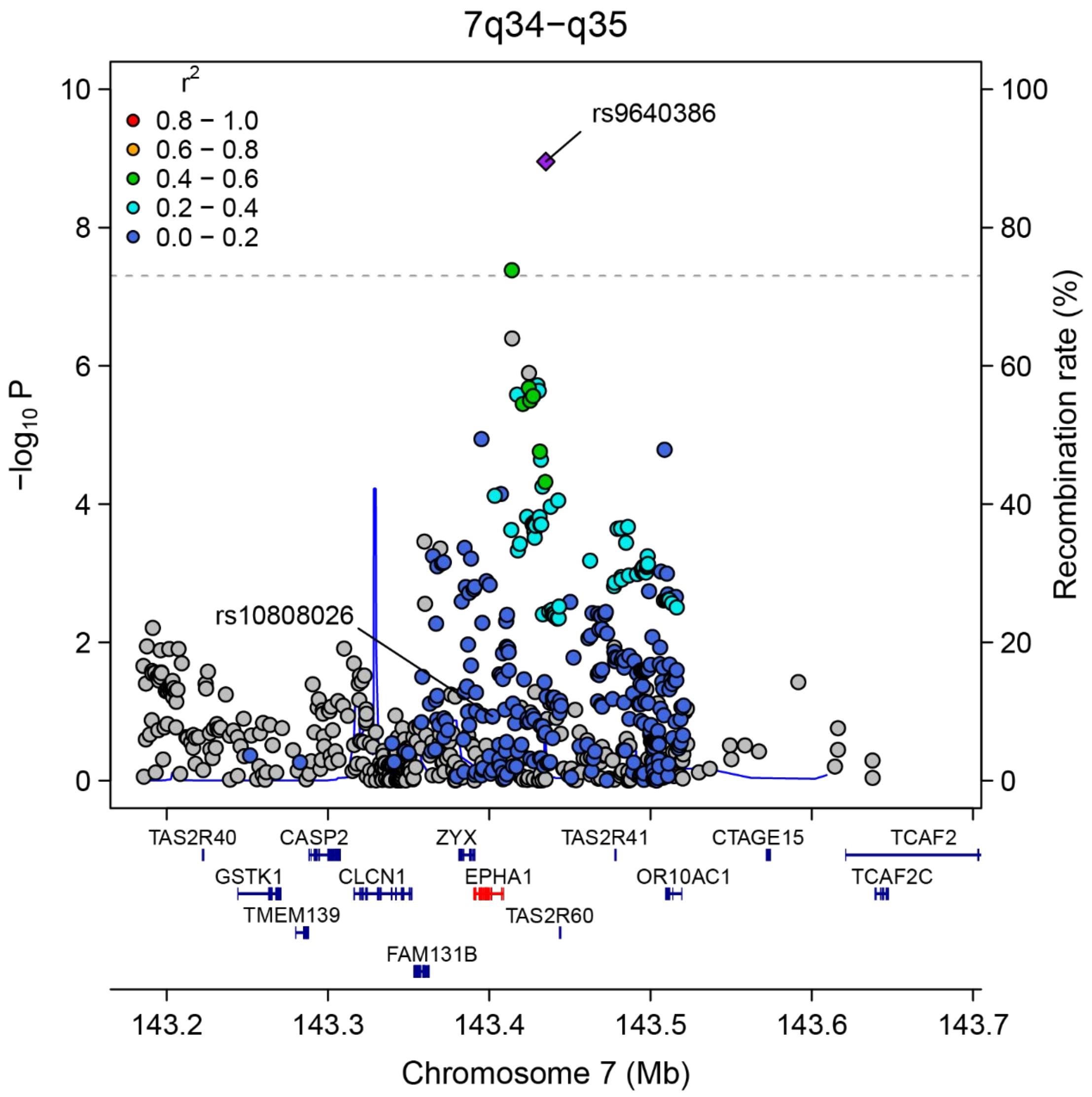

Supplemental Figure 14

LocusZoom for 19q13.32 near *APOC1*.

X-axis: Position on GRCh38. **Left Y-axis:** -log10P-values corresponding to data points. **Right Y-axis:** recombination rate, corresponding to the blue line. Points are color-coded with evidence for linkage disequilibrium (r^2) in the 1000 Genomes European reference sample. The candidate gene from prior GWAS is highlighted in red.

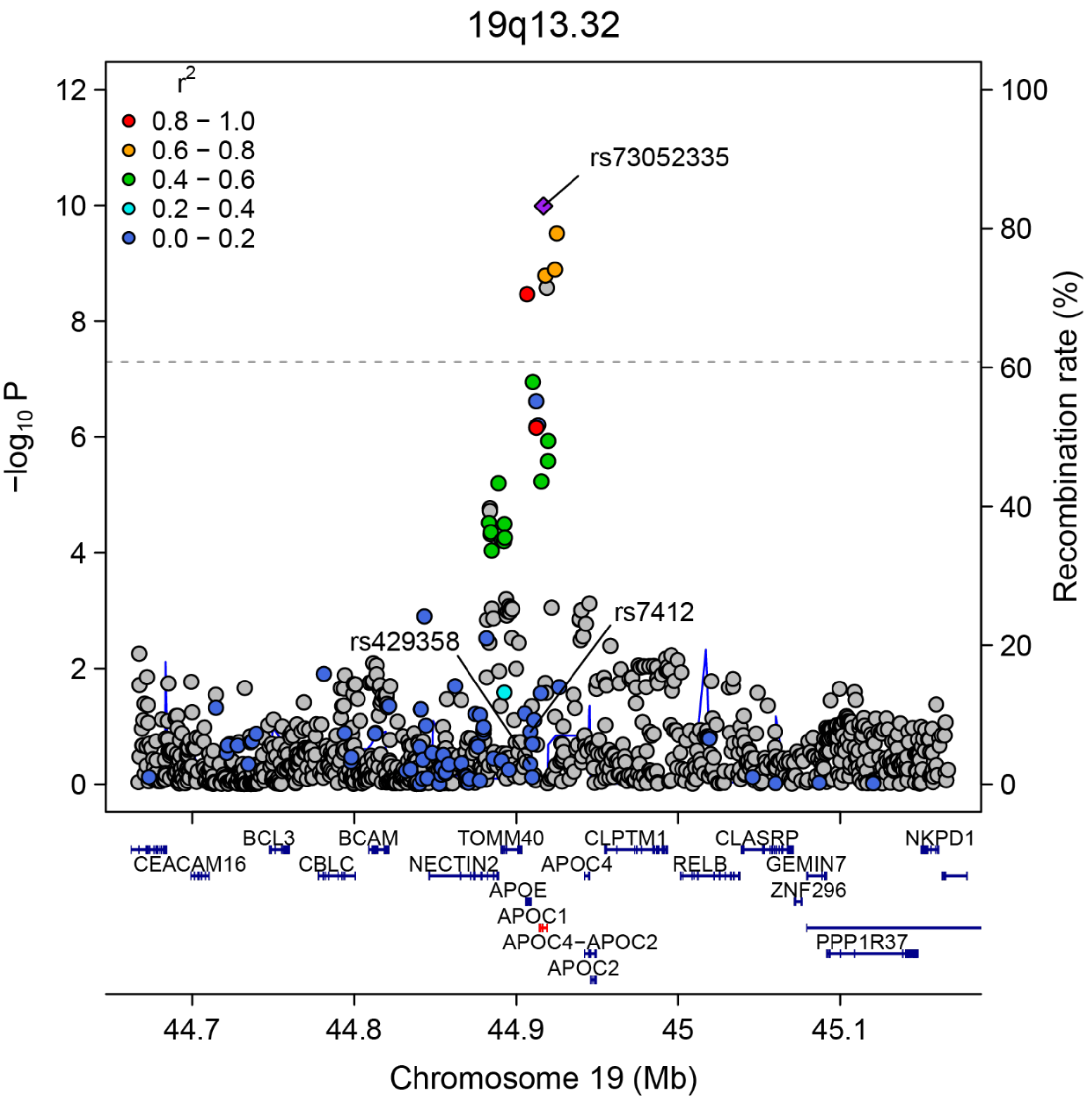

Supplemental Figure 15

Genome-wide association study for age-at-onset of Alzheimer's disease, adjusting for sex, population structure, and relatedness.

Top: The discovery GWAS of 9,111,204 variants and 22,044 participants identified significant evidence of association at eight well-established AD risk loci near *APOE*, *ABCA7*, *BIN1*, *CR1*, *MS4A4A*, *PICALM*, *PILRA*, and *SHARPIN* (λ = 1.03, LD score intercept = 1.04, **Supplemental Table 1**). **Middle:** The replication GWAS (λ = 1.04, LD score intercept = 1.05) supported each signal from the discovery GWAS, sharing the same direction of effect and significant p-values ($p < 0.05/8$ loci), except for *ABCA7* whose associated alleles did not pass imputation QC in the replication data (**Supplemental Table1**). Variants at known AD risk loci near *APOE*, *BIN1, CR1*, and *MS4A4A,* reached genome-wide significance, as well as a novel signal on 12p13.2 (rs4763849, alternate allele frequency [AAF] = 0.369, β = -0.041, p = 1.18E-08). **Bottom:** Meta-analysis identified 13 genome-wide significant associations, adding signals at known AD GWAS loci near *CASS4*, *CD2AP*, *EPHA1*, *MAF*, and *SCIMP* as well as a new signal on 9q21.32 (**Supplemental Table 2**; λ = 1).

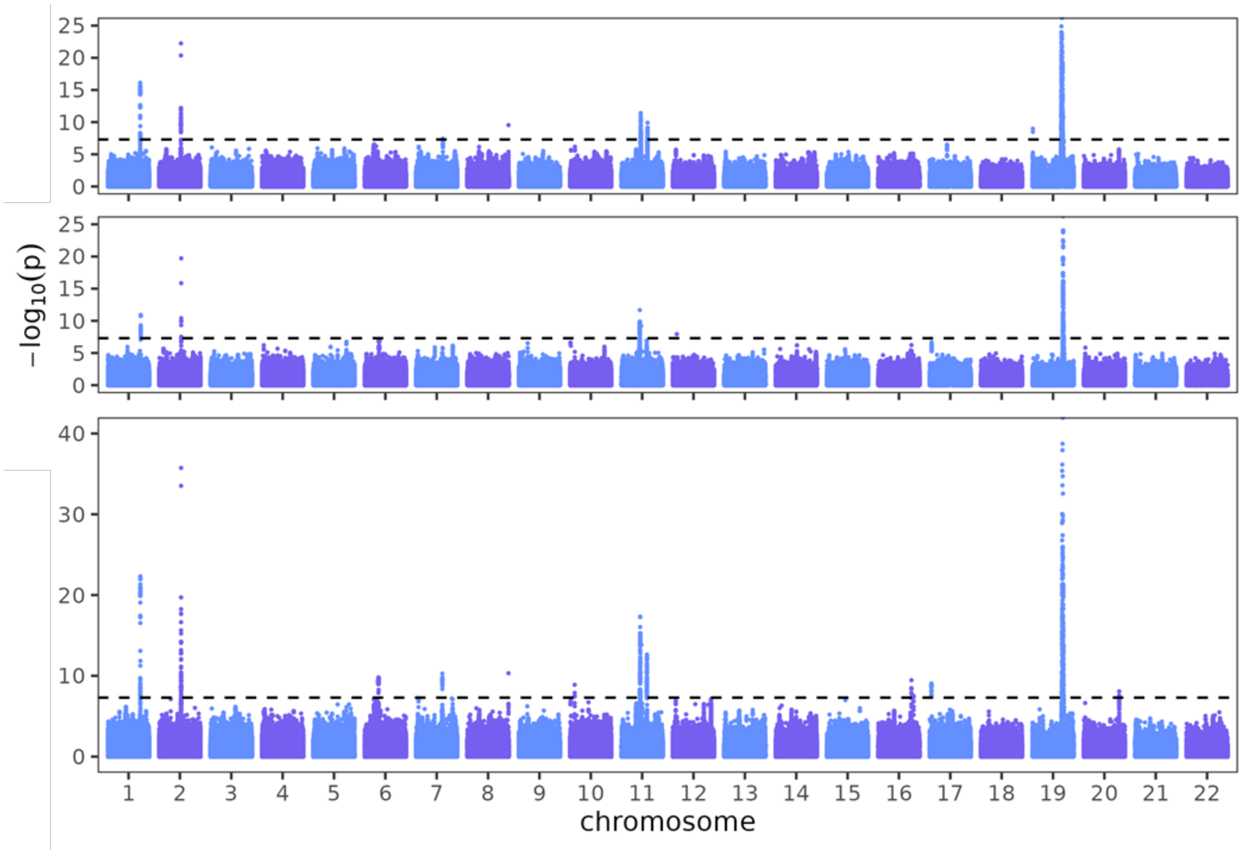

Supplemental Figure 16

Genome-wide association study for age-at-onset of Alzheimer's disease, adjusting for only population structure and relatedness.

Top: The discovery GWAS of 9,111,559 variants and 21,736 participants identified significant evidence of association at seven well-established AD risk loci near *ABCA7*, *APOE*, *BIN1*, *CR1*, *MS4A4A*, *PICALM*, and *SHARPIN* (λ = 1.03, LD score intercept = 1.04. **Supplemental Table 3**). **Middle:** The replication analysis of 8,634,132 variants and 19,483 participants supported each of the discovery GWAS signals except for *ABCA7* as the corresponding variant did not pass QC (λ = 1.04, LD score intercept = 1.05). The replication GWAS also identified significant evidence of association at two new loci: rs1320688 on 11q13.1 (alternate allele frequency [AAF] = 0.856, β = -0.068, *p* = 7.15E-10) and rs4763849 on 12p13.2 (AAF = 0.369, β = -0.041, *p* = 1.66E-08). **Bottom:** Meta-analysis of the discovery and replication GWAS identified 14 genome-wide significant associations (λ = 1), including known AD risk loci near *APOE*, *BIN1*, *CASS4*, *CD2AP*, *CR1*, *MAF*, *MS4A4A*, *PICALM*, *PILRA*, *SCIMP*, *SHARPIN*, and *UMAD1* while adding two loci on 2p16.1 and 12q24.31 (λ = 1, **Supplemental Table 4**).

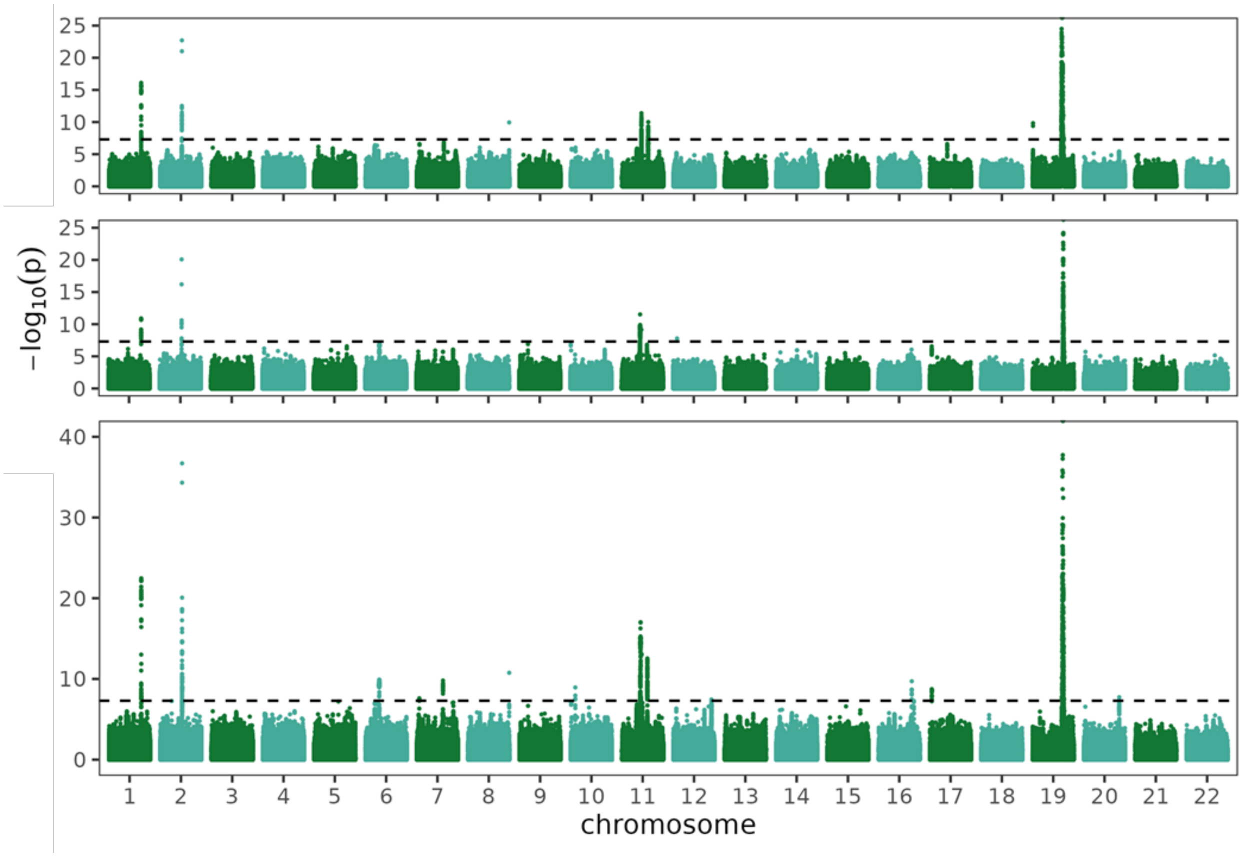

Supplement: Supplementary file 1 — Supporting information [file ALZ-21-e70489-s002.pdf]
